# BD-2 and BD-3 increase skin flap survival in a model of ischemia and *Pseudomonas aeruginosa* infection

**DOI:** 10.1038/s41598-019-44153-y

**Published:** 2019-05-27

**Authors:** Diogo Casal, Inês Iria, José S. Ramalho, Sara Alves, Eduarda Mota-Silva, Luís Mascarenhas-Lemos, Carlos Pontinha, Maria Guadalupe-Cabral, José Ferreira-Silva, Mário Ferraz-Oliveira, Valentina Vassilenko, João Goyri-O’Neill, Diogo Pais, Paula A. Videira

**Affiliations:** 1 Anatomy Department, NOVA Medical School, Universidade NOVA de Lisboa, Lisbon, Portugal; 2 Plastic and Reconstructive Surgery Department and Burn Unit, Centro Hospitalar de Lisboa Central – Hospital de São José, Lisbon, Portugal; 3 UCIBIO, Departamento de Ciências da Vida, Faculdade de Ciências e Tecnologia, Universidade NOVA de Lisboa, Lisbon, Caparica Portugal; 4 CEDOC, NOVA Medical School, Faculdade de Ciências Médicas, Universidade NOVA de Lisboa, Lisbon, Portugal; 5 Pathology Department, Centro Hospitalar de Lisboa Central – Hospital de São José, Lisbon, Portugal; 6 LIBPhys, Physics Department, Faculdade de Ciências e Tecnologias, Universidade NOVA de Lisboa, Lisbon, Caparica Portugal; 7 CDG & Allies- Professional and Patient Association International Network (PPAIN), Lisbon, Caparica Portugal; 8 Molecular Microbiology and Biotechnology Unit, iMed, ULisboa, Faculty of Pharmacy, Universidade de Lisboa, Lisbon, Portugal; 9 INESC MN – Microsystems and Nanotechnologies, Instituto Superior Técnico, Universidade de Lisboa, Lisbon, Portugal

**Keywords:** Bacterial infection, Experimental models of disease, Infection

## Abstract

The main aim of this work was to study the usefulness of human β-defensins 2 (BD-2) and 3 (BD-3), which are part of the innate immune system, in the treatment of infected ischemic skin flaps. We investigated the effect of transducing rat ischemic skin flaps with lentiviral vectors encoding human BD-2, BD-3, or both BD-2 and BD-3, to increase flap survival in the context of a *P*. *aeruginosa* infection associated with a foreign body. The secondary endpoints assessed were: bacterial counts, and biofilm formation on the surface of the foreign body. A local ischemic environment was created by producing arterialized venous flaps in the left epigastric region of rats. Flaps were intentionally infected by placing underneath them two catheters with 10^5^ CFU of *P*. *aeruginosa* before the surgical wounds were hermetically closed. Flap biopsies were performed 3 and 7 days post-operatively, and the specimens submitted to immunohistochemical analysis for BD-2 and BD-3, as well as to bacterial quantification. Subsequently, the catheter segments were analyzed with scanning electron microscopy (SEM). Flaps transduced with BD-2 and BD-3 showed expression of these defensins and presented increased flap survival. Rats transduced with BD-3 presented a net reduction in the number of *P*. *aeruginosa* on the surface of the foreign body and lesser biofilm formation.

## Introduction

Multi-resistant bacteria are on the rise all over the world, leading many to proclaim an imminent post-antibiotic era, in which common infections and surgeries could become life threatening conditions^1–4^. For instance, several authors have shown that a leg ulcer lasting for one month will have on average at least one isolated multi-drug resistant organism at that time^5^. Hence, it comes as no surprise that multiple new strategies to tackle bacterial infections are actively being sought^1–4,6^ and alternatives to conventional antibiotics are direly needed^7^.

In this context, antimicrobial peptides (AMPs) become particularly appealing as a new way to target one of the last “Achilles’ heels” of bacteria^4,6,8–14^. AMPs are small cationic peptides that have pleotropic bactericidal effects. They are divided in multiple chemical classes, of which the most studied and populated are the cathelicidins, the defensins, the histatins, and the dermcidins^6^. Defensins have been indicated as one of the most promising AMP classes for potential therapeutic used based on *in vitro* essays^14–16^. Defensins, similarly to other AMPs, act mainly by disrupting the structure of gram-positive and gram-negative bacterial cell membrane. These AMPs also inhibit bacterial DNA replication, transduction and translation, disturbing bacterial homeostasis (Supplementary Fig. 1). The resulting byproducts trigger the activation of the complement system and inflammatory processes that further help clearing bacterial infections (Supplementary Figs 1 and 2)^8,11,17^. Moreover, it has been shown that due to its peculiar action in the cell membrane, seldom are bacteria able to develop resistance to defensins^6,18,19^.

Defensins, of which 15 isoforms are currently identified in humans, are remarkably widely secreted in multiple epithelia, leucocytes and platelets (Supplementary Fig. 2)^15,20,21^. Apart from their role in the innate immunity, it has been recognized that they are instrumental in immune regulation and to initiate, mobilize, and amplify acquired immunity^6^.

Among the most commonly encountered multi-resistant bacteria, *Pseudomonas aeruginosa* stands out as one of the major culprits of nosocomial infections worldwide, being associated with significant morbidity, mortality, and increased health costs. *P*. *aeruginosa* frequently causes antibiotic-refractory infections of prosthetic material, due to its ability to produce biofilms, and to its intrinsic, acquired and adaptive resistance mechanisms to multiple antibiotics^2,22,23^. Noteworthy, *P*. *aeruginosa* is known to thrive in poorly perfused tissues, as in ischemic wounds or limbs, such as those of many diabetic patients, as well as in chronic wounds and/or around prosthetic material^5,7,24,25^.

Although numerous AMPs have shown microbicidal activity against *P*. *aeruginosa in vitro*^17,26–28^, as far as the authors could determine, there are no studies using β-defensins (BDs) to treat *P*. *aeruginosa* infections in *in vivo* models^27,28^. This is unfortunate, since BDs have shown to be efficient against multi-resistant *P*. *aeruginosa in vitro*^27,28^. Moreover, amongst BDs, BD-2 has been shown to be particularly effective against other Gram-negative bacteria and some fungi, although relatively less potent against Gram-positive bacteria. Furthermore, BD-3 is reportedly a powerful antimicrobial agent with a broad range of activity toward yeast, Gram-negative and Gram-positive bacteria, including the vancomycin-resistant *Enterococcus faecium*^26,27^.

Despite these interesting *in vitro* results, there are several reports postulating that defensins, being cationic, probably have hampered bactericidal activity *in vivo* due to the presence of neutralizing anionic compounds in living tissues^27,29,30^.

Hence, the main aim of this work was to study the usefulness of transducing an ischemic skin flap in the rat with two human BDs (BD-2 and BD-3) to increase flap survival in the context of a *P*. *aeruginosa* infection associated with a foreign body. The secondary endpoints assessed were: reduction in bacterial counts, reduction in biofilm formation, and increase in rat survival rates. Interestingly, we observed that BD-2 and BD-3 increased skin flap survival in our model. Moreover, rats transduced with BD-3 presented a net reduction in the number of *P*. *aeruginosa* on the surface of the foreign body and lesser biofilm formation.

## Materials and Methods

**Figure 1:**
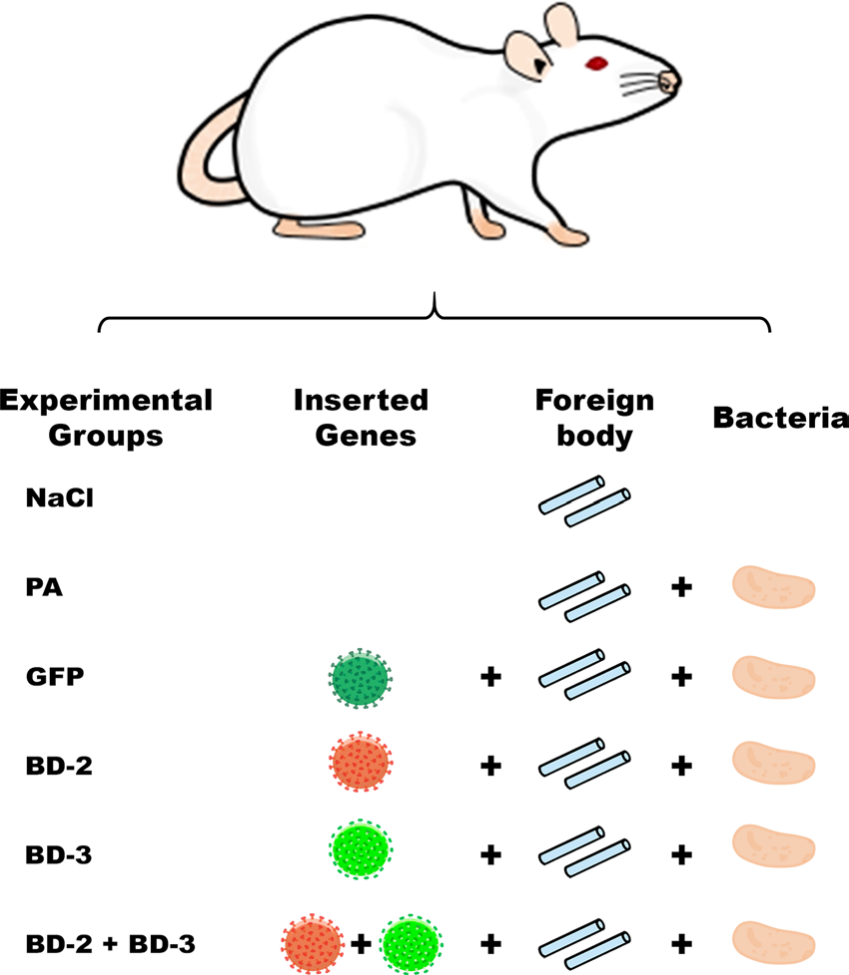
Diagram illustrating the experimental groups used in this work. In all groups, flaps were intravascularly injected with a 100-µl solution of recombinant rat Vascular Endothelial Growth Factor-A that was left to act for 90 min. In the NaCl group, before closing the surgical wounds, one milliliter of a 0.9% sodium chloride solution was instilled under the flap into the vicinity of the silicone catheter segments. In the PA group, one milliliter of a 0.9% sodium chloride solution containing 10^5^ CFU *Pseudomonas aeruginosa* was instilled under the flap into the vicinity of the silicone catheter segments. In the GFP, BD-2, BD-3 and BD-2 + BD-3 groups, besides the procedure described for the PA group, a solution containing a lentivirus coding for Green fluorescent protein, human β-defensin 2, human β-defensin 3 and human β-defensins 2 and 3 was injected in the flap’s vessels, respectively.

**Figure 2:**
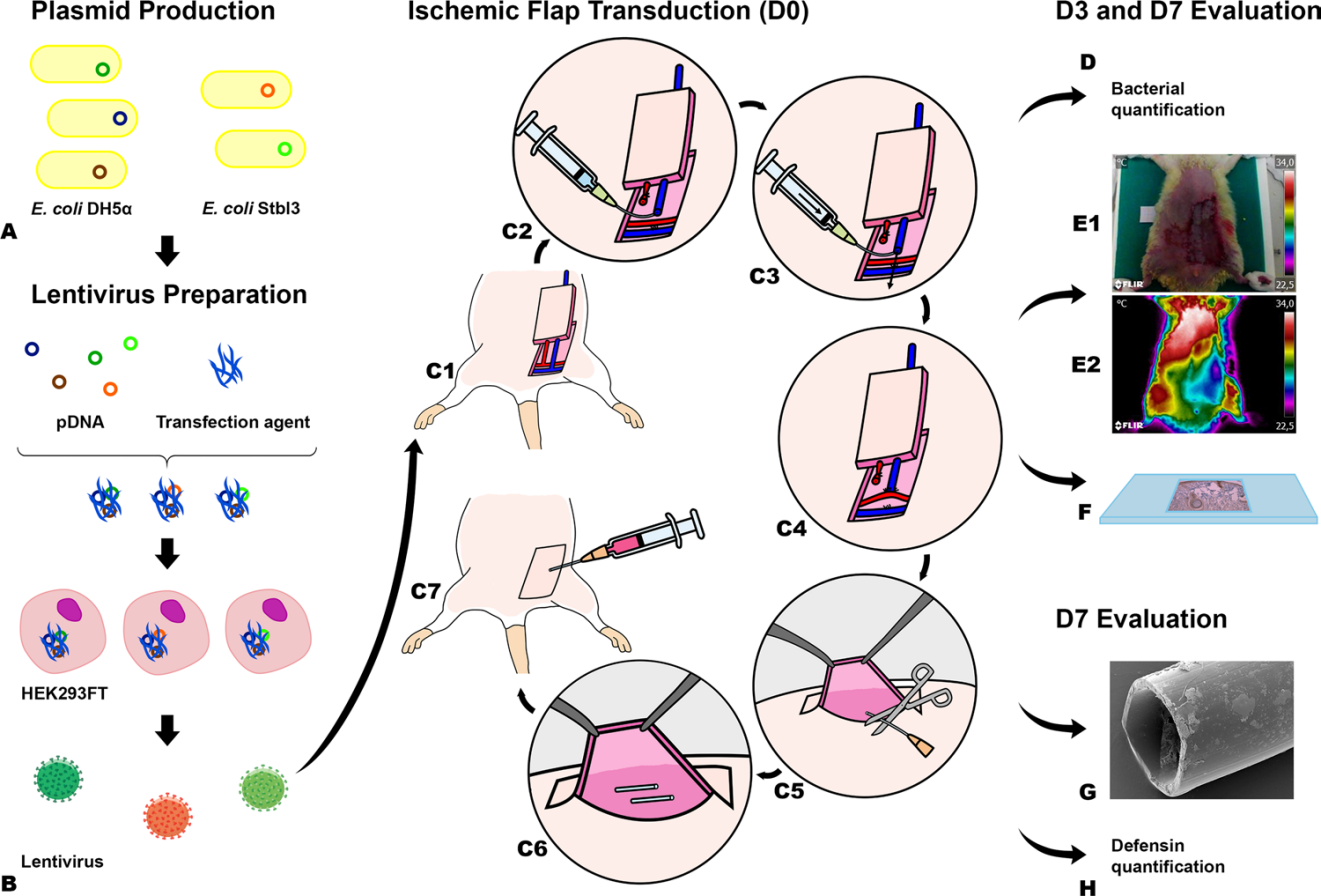
Diagram illustrating the main steps in the production of the rodent model of ischemia, *Pseudomonas aeruginosa* infection associated with a foreign body, lentiviral delivery of antimicrobial peptides, and evaluation of tissue necrosis. (**A**) Plasmid production by using transformed *Escherichia coli* (*E*. *coli*). (**B**) Lentivirus construction using HEK293FT cells. From (C1) to (C7) the steps involved in the production of a model of foreign body infection associated with an ischemic fasciocutaneous flap are depicted. (**D**) Flap biopsies were collected on the third and seventh day postoperatively to quantify bacteria. (**E**) Flap survival and perfusion was assessed by clinical inspection (E1) and with resort to direct infrared thermography (E2) and to histological analysis (**F**). (**G**) Bacterial numbers and distribution on the surface of foreign bodies retrieved 7 days postoperatively were determined using scanning electron microscopy. (**H**) Flap transduction with the human β-defensin 2 (BD-2) and/or β-defensin 3 (BD-3) genes was evaluated by immunohistochemical evaluation of flap biopsies 3 and 7 days after surgery. VEGF, Vascular Endothelial Growth Factor.

Figures 1 and 2 summarize the experiments done in this work.

### Bacterial growth

All bacteria were inoculated in Luria-Bertani (LB) broth and grown at 37 °C on a rotary shaker at 250 rpm, overnight. Exceptions were performed and are properly indicated.

#### Plasmids production

Competent *Escherichia coli* DH5α were transformed by heat-shock with pMD2.G and psPAX vectors, while competent *E*. *coli* Stbl3 were transformed with pLenti6.BD-2 and pLenti6.BD-3. Posteriorly, bacteria were grown in LB media, at optimal temperature in ampicillin presence, at 37 and 30 °C, respectively.

#### *Pseudomonas aeruginosa* – infection model

*P*. *aeruginosa* CECT 110^+^ strain (also known as *P*. *aeruginosa* ATCC® 10145™, which is widely used for antibiotic testing and for microbiological assays since 1947) was used to infect rats^31–33^. This strain was grown until the mid-exponential phase and it was serially diluted in 0.01 M PBS at a OD_600nm_ of 0.0001 corresponding to 1.0 × 10^5^ CFU/ml. At all times, to confirm *P*. *aeruginosa* CFUs (colony forming units), spreadings of bacteria were performed in LB agar dishes and colonies were counted after overnight incubation at 37 °C.

Before performing the antimicrobials assays in the animal model of ischemia, tests were conducted to determine the ideal bacterial concentration. The concentrations initially used were: 10^3^; 10^4^; 10^5^; 10^6^ and 10^7^ CFU/ml. We selected the concentration of 10^5^ CFU/ml, since this concentration systematically resulted in clinically significant surgical site infection and partial flap necrosis in our model. Higher concentrations led to complete or nearly complete flap necrosis (10^6^ CFU/ml; n = 2) and animal’s death (10^7^ CFU/ml; n = 3), whereas lower concentrations (10^3^ and 10^4^ CFU/ml; n = 3 for each concentration) did not produce clinically recognizable infection.

### Lentivirus preparation

The defensin beta 4 A (*DEFB4A*) and defensin beta 103 A (*DEFB103A*) cDNA sequences, which code for the BD-2 and BD-3 proteins, respectively, were synthetized by GenScript Corporation® (New Jersey, USA) and cloned into pcDNA ENTR BP, using *Xho*I/*EcoR*I (*DEFB4A*) and *Sal*I/*Kpn*I (*DEFB103A*). This expression vector was generated by inserting a polylinker, previously chemically synthetized, containing several restriction sites into pcDNA6.2 GW/Em-GFP, a mammalian expression Gateway® (Invitrogen) previously digested with DraI/XhoI. The defensins coding sequences were transferred into pLenti6 (Invitrogen®) by LR recombination^34^.

Lentivirus particles were produced according to ViraPower™ Lentiviral Expression Systems (ThermoFisher^®^) recommendations, using HEK293FT cells (Invitrigen^TM^, ThermoFisher^®^, R700-07). Recombinant BD-2 and BD-3 expression in HEK293 was confirmed by RT-PCR. The total RNA of cells lines was extracted using the GenElute™ Mammalian Total RNA Miniprep Kit (Sigma-Aldrich^®^), according to the manufacturer’s instructions. In order to remove any genomic DNA contamination, a RNase-Free DNase Set (Qiagen®) was used. The SuperScript^®^ III First-Strand Synthesis System (Invitrogen™) was used to perform the reverse transcription, following the manufacturer’s instructions. PCR amplification was performed using primers for the genes of BD-2 and BD-3 (Supplementary Table 1).

### Ethics statement

All procedures involving animal subjects were approved by the Institutional Animal Care and Use Committee and Ethical Committee at our institution (08/2012/CEFCM). All *in vivo* studies involving rats were carried according or exceeding the recommendations in the Guide for Proper Conduct of Animal Experiments and Related Activities in Academic Research and Technology^35^.

### Animals

One hundred and two male adult Wistar rats weighing 250 to 350 grams were used.

Of the 102 operated rats, 19 died in the immediate postoperative period (<24 hours), and were not included in the study. The following number of rats was included in each experimental group: 12 in the NaCl control group; 13 in the *P*. *aeruginosa* group; 14 in the GFP group; 15 in the BD-2 transduced group; 15 in the BD-3 transduced group, and 14 in the BD-2 + BD-3 transduced group. There were no statistically significant differences between the different experimental groups regarding survival throughout the experiment.

All the animals were housed under standard environmental conditions and given nothing by mouth six hours before surgical procedures. No antibiotic prophylaxis was given.

Rats were anesthetized with a mixture of ketamine (5 mg/kg) and diazepam (0.25 mg/kg) given intraperitoneally. The depth of anesthesia was evaluated by toe pinch and by observance of respiration rate throughout the entire procedure^36–39^. Supplementary doses of the anesthetic mixture were provided throughout the surgical procedures as needed^40^. An ophthalmic gel was applied over the anterior surface of the eyes to avoid corneal abrasion. The hair over the ventral surface of the abdomen was removed with a depilatory cream (Veet®). After hair removal, the depilatory cream was cleaned from the abdomen with warm saline. After shaving the abdomen and placing the animals on the operation table, body temperature was recorded with a rectal thermometer and the rats were kept on a heating pad (Skaldo, Ardes ™), in order to maintain a constant body temperature. A substantial amount of an alcoholic solution (Cutasept F®, Hartmann™) was sprayed over the operative site. The product was left in the operative site for at least 15 seconds. Application was repeated 3 times. After the last application, the disinfectant solution was left in contact with the operating field for at least 2 min before proceeding with the surgery. All surgical procedures were performed in strict sterility conditions.

### Surgical Model

Succinctly, a variant of the rat abdominal arterialized venous flap was used as model of ischemic flap (Fig. 2)^41,42^. This flap was transduced with an *ex vivo* infusion of a 100 µl solution containing approximately 4.7 × 10^9^ plaque-forming-units of recombinant lentiviruses coding for Green Fluorescent Protein (GFP) or BD-2 and/or BD-3^43,44^. This model was combined with an adapted version of the rat foreign body infection model (Figs 1 and 2**)**^45^.

In all animals, under a surgical operating microscope, a 5 cm long and 3 cm wide fasciocutaneous flap was raised on the left side of the rat’s abdomen^42^. Cranially, the flap was connected exclusively to the skeletonized lateral thoracic vein^46^. This vein was temporarily clamped. Caudally, the superficial caudal epigastric artery was ligated with an 8/0 Nylon suture. The superficial caudal epigastric vein (SCEV) was cannulated with a 27-gauge ophthalmic cannula (BD Bioscience™). It was then perfused with a 100-µl solution whose composition varied amongst the experimental groups (Figs 1 and 2**)**. In all cases the solution contained 5 µg of recombinant rat Vascular Endothelial Growth Factor A (VEGF; Immunotools®) in DMEM, in order to increase transduction efficiency^43^. The composition of the solution injected in the flap’s venous system was as follows: **NaCl Group** (VEGF); **PA Group** (VEGF); **GFP Group** (VEGF + GFP coding lentivirus); **BD-2 Group** (VEGF + BD-2 coding lentivirus); **BD-3 Group** (VEGF + BD-3 coding lentivirus); **BD-2 + BD-3 Group** (VEGF + BD-2 + BD-3 coding lentivirus) (Fig. 1). After injection, SCEV was immediately clamped for 90 min, in order to leave the injected solution in contact with the flap’s vascular system^43^.

Subsequently, vascular clamps were removed and the SCEV was arterialized by connecting it to the femoral artery. In order to achieve this, the SCEV was cut from the femoral vein with a 1-mm long cuff of adjacent femoral vein tissue. The ostium in the femoral vein was closed with a continuous Nylon 11/0 suture. The same suture line was used to perform a side-to-end arteriovenous anastomosis between the SCEV and the ventral flank of the femoral artery through a 1-mm long ostium previously created in the latter vessel. Interrupted stitches were used for this anastomosis. The lateral thoracic vein was preserved cranially to ensure outflow from this arterialized venous flap^42^.

Two 1-cm segments of sterile 14-gauge silicone catheters (Mediplus™) were placed in the central aspect of the surgical wound. Surgical wounds were closed with a running 5/0 Nylon suture. Just before closing the lateral aspect of the skin wound, 1-ml of a 0.9% sodium chloride solution was instilled under the flap into the vicinity of the silicone catheter segments using an 18-gauge silicone catheter (Mediplus™). In all groups except the NaCl group, the latter solution contained 10^5^ CFU of *P*. *aeruginosa*. The running suture was closed immediately after injection, in order to avoid any spilling to the area adjacent to the flap (Fig. 2).

No anticoagulants or antibiotics were administered pre, intra or postoperatively. All surgical procedures were performed by the same surgeon (D.C.), in order to minimize inter-surgeon variability.

### Thermography

One hour after flap reperfusion, with the rat still under anesthesia, the rat’s abdomen was submitted to infrared thermography with a FLIR® E6 camera placed 25 cm above the abdomen. This evaluation intended to confirm the flap’s relative ischemia compared to the contralateral side^42^. Rats were placed on their backs for 10 min prior to this evaluation. Thermographic measurements were made at a constant room temperature (22 °C) and humidity (50%)^42,47^.

### Post-operative care and assessment

After thermographic evaluation was completed, a transparent Tegaderm™ (3 M Deustschland GmbH®) transparent film dressing was applied over the flap and adjacent skin. Following surgery, rats were kept in solitary rat cages and offered rat chow and water *ad libitum*.

Rats were assessed daily by the same blinded researcher, in order to reduce inter observer bias and variability^48^. The following parameters were evaluated: animal wellbeing, flap viability, and presence of complications.

On the third and seventh days postoperatively, rats were anesthetized as described above. Objective measurement of flap survival was performed on these days based on digital photographs, which were later analyzed by a blinded observer using the free Image J® software^40,49^. The total flap area and the necrotic flap area were determined for each flap. The necrosis rate for each rat was defined by the proportion of the total flap area that was occupied by the necrotic flap area, being expressed as a percentage of the total flap surface area^50^.

After disinfecting the rat’s abdomen as detailed above, on the third postoperative day, a sample measuring approximately 1 cm in length and 0.5 cm in width was surgically excised from the most lateral aspect of the caudal third of the viable flap. These specimens were subjected to histological analysis, as well as bacterial counts by both culture and real-time PCR. The integumentary defect created by the biopsy was submitted to hemostasis and closure with a continuous 5/0 Nylon suture taking part of the integumentary redundancy in this region. A transparent Tegaderm™ (3 M Deustschland GmbH®) transparent film dressing was again applied over the flap and adjacent skin.

On the seventh day after the first surgery, the lateral aspect of the flap was elevated, thus exposing the underlying catheter segments. These were then carefully removed, taking care not to touch the rat’s skin and immediately immersed in the fixative solution.

The flap was then removed for histological analysis, as well as bacterial counts by culture and real-time PCR (Supplemental Tables 2 and 3).

Rats were euthanized on the seventh postoperative day by exsanguination under general anesthesia after cutting the common carotid arteries and the external jugular veins^51^.

### Evaluation of flap transduction by fluorescence microscopy

Four rats were submitted to the procedures described above for the GFP group with the exception that no bacteria were instilled. Seven days after the surgery, the skin flap was harvested, fresh-frozen and observed under the fluorescence microscope. Two rats were submitted to an analogous the procedure, but no lentiviruses were used to transduce the flaps in these animals. Fluorescence microscopy images were obtained in the same manner, using the same image acquisition parameters, by two blinded observers.

### Quantification of BD-2 and BD-3 expression by quantitative real-time PCR

Total RNA from flap biopsies weighing (45–50 mg) collected on the 7^th^ day postoperatively was extracted using the GenElute^TM^ Mammalian Total RNA Miniprep Kit (Sigma-Aldrich). The RNase-Free DNase Set (Qiagen®) was used to eliminate genomic DNA, according to the manufacturer’s instructions. RNA concentrations were measured and only samples with A260/A280 ratios between 1.8 and 2.1 were considered further. Five hundred nanograms of total RNA was reverse transcribed with random primers using the High Capacity cDNA Reverse Transcription Kit (Applied Biosystems^TM^), according to the manufacturer’s instructions. Real-time PCR was performed in a Rotor-Gene 6000 (Corbett Life Science) using TaqMan® Fast Universal PCR Master Mix (Applied Biosystems^TM^) [Supplemental Table 2]. Each reaction was performed in triplicate. Thermal cycling conditions were 95 for 20 seconds followed by 55 cycles of 95 for 3 seconds and 60 for 30 seconds. The gene expression was normalized to the endogenous control β-actin, which is known to have significant basal expression^52^. Gene relative expression was calculated using the 2^−ΔCT^ × 1000 formula, an adaptation of the 2^−ΔΔCt^ method described by Livak and Schmittgen^53–55^. This allowed us to calculate the number of mRNA molecules of the gene of interest per 1000 molecules of the endogenous control (β-actin).

### Viable bacterial cell counts

Biopsies were collected in sterility and cut in small pieces (between 0.05 and 0.10 mm of maximum diameter). Next, they were macerated in 0.9%(w/v) NaCl (Merck^®^) at 100 mg/mL concentration for 5 min with a Pellet pestle (Sigma-Aldrich^®^). Serial dilutions were performed from the supernatant in 0.9%(w/v) NaCl (between 10^−1^ and 10^−5^) and were spread on LB dishes in duplicated. The dishes were incubated at 37 °C during 14 h followed by quantification of CFUs. The CFUs counted were verified by running a series of tests, namely, Gram stain (Merck^®^); MacConkey growth (Carl Roth^®^) and *Oxidase* test (BioMérieux^®^) according to the manufacturers’ instructions.

To certify the inoculation dose of *P*. *aeruginosa*, the effective average bacterial inoculation dose under the flap was determined by bacterial growth and found to be (9.735 ± 0.120) X 10^4^ CFU, which was similar to the initial inoculation concentration of 10^5^ CFU/ml. There were no statistically significant differences between the initial bacterial inoculum in the different experimental groups.

### *P*. *aeruginosa* quantification by real-time PCR

Biopsies were collected in sterility, were immediately immersed in RNAlater^®^ (Sigma-Aldrich^®^), and incubated overnight at 4 °C. On the following day, the biopsies were cut in small pieces (between 0.05 and 0.10 mm) and stored at −80 °C.

Forty-five to 50 mg of excised tissue was used to extract genomic DNA by NZY Tissue gDNA Isolation Kit (Nzytech^®^) according to the manufacturer’s instructions.

To quantify *P*. *aeruginosa*, universal primers were used for 16 S rDNA (Supplementary Table 3)^56^. The real-time PCR was performed using Applied Biosystems 7500 real-time PCR System (Thermo Fisher Scientific^®^). The reaction mixture (10 µl) contained 2x SYBR Green PCR Master Mix (Thermo Fisher Scientific^®^), 0.1 nmol/µl of *forward* and *reverse* primers each and 5 ng/µl of *P*. *aeruginosa* gDNA.

Thermo-cycling program was 40 cycles of 95 °C for 20 s, 60 °C for 30 s and 72 °C for 40 s with an initial cycle at 50 °C for 2 min and 95 °C for 10 min. Next, a dissociation curve was performed at 95 °C for 15 s and a range between 60 to 95 °C.

### Histological processing

Flap biopsies were collected from the lateral aspect of the surgical flap of the rat 3 and 7 days postoperatively, as described above. Specimens were incubated in 10% (v/v) Formalin. After paraffinization (VWR^®^), specimens were cut on a microtome (Leica^®^) as 3 µm thick slices. These slices were stained with Hematoxylin-Eosin (HE) and Masson’s Trichrome (MT), according to the manufacturers’ indications^57,58^. For immunohistochemical staining with anti-BD-2 and -BD-3, slices were prepared in an analogous fashion, although they were cut 4 µm thick.

#### Immunohistochemistry processing for BD-2 and BD-3

Imunohistochemistry was performed by BenchMark ULTRA - Automated IHC/ISH slide staining system (Ventana^®^, Roche^®^). Summarily, the slides were heated at 80 °C for 15 min, deparaffinized with EZ prep (Ventana^®^, Roche^®^) for 8 min. Antigen retrieval was performed with an ULTRA CC2 (Ventana®, Roche®) at 95 °C for 8 min (for BD2 staining) and ULTRA CC1 (Ventana®, Roche®) at 95 °C for 20 min (for BD3 staining). Endogenous peroxidase was blocked with 3% (v/v) Hydrogen Peroxide (UltraView Universal DAB Inhibitor, Ventana®, Roche®) for 4 min. The following primary monoclonal antibodies were used at 37 °C: anti-BD-2 (β-defensin 4 (L13-10-D1): sc-59496; Santa Cruz Biotechnology, inc.®) [1:50] for 40 min; and anti-BD-3 (BD-3 human (SRP4524), Sigma Aldrich®) [1:2], for 32 min. Amplification was accomplished with the UltraView Universal HRP Multimer (Ventana®, Roche®) for 8 min. Revelation was performed with the UltraView Universal DAB Chromogen and the UntraView Universal DAB H_2_O_2_ for 8 min (Ventana®, Roche®) followed by intensification with the UltraView Universal DAB Copper (Ventana®, Roche®) for 4 min. The nuclear contrast was performed with Hematoxylin and Bluing (Ventana®, Roche®) for 4 min each.

Finally, the slides were washed in water and detergent, dehydrated with increasing concentrations of ethanol (75, 90 and 99% (v/v)) for 1 min each, cleared in Xylene (VWR®) and mounted with synthetic mounting medium (Quick-D M-Klinipath®).

### Catheter processing for SEM

The catheters were introduced in mounting pins to incubate at 4 °C, overnight in a solution of 2%(v/v) Glutaraldehyde (Sigma-Aldrich®) in 0.05 M Sodium Phosphate Buffer (pH = 7.4) (Merck^®^) to fixate the specimens. Catheters were washed 3 times in 0.15 M Sodium Phosphate Buffer (pH = 7.4). Post fixation was performed in a solution of 1%(v/v) Osmium Tetroxide (Sigma^®^) in 0.12 M Sodium Cacodylate Buffer (pH = 7.4) (Merck^®^) during 2 h at room temperature. The catheters were rinsed in deionized water and were dehydrated in increasing concentrations of ethanol for analysis (10, 25, 40, 60, 95, 100%) (Merck^®^) for 20 min for each incubation. Next, they were maintained in Acetone Pro-Analise (Merck^®^) overnight. Critical Point (Polaron® E3100) was executed within a range of pressure and temperature of 78–80 Bar and 28–30 °C, respectively. Three purges using CO_2_ (Gains^®^) were performed with an interval of 1 h between each. SPI Flash-Dry™ Silver Paint (SPI Supplies^®^) was used to mount the catheters to the stubs (Agar Scientific^®^). Catheters were metalized (Polaron^®^ SC502) with Gold in the presence of Argon (Airliquide^®^) at a pressure between 1 and 4 Pa and a current of 0.015 A. Four metallization cycles were performed. The specimens were examined with two scanning electron microscopes: a JEOL JSM-5410, with acceleration voltage of 0.015–0.030 V, for quantification purposes, and a JEOL JSM-7001F, with acceleration voltage of 0.015–0.030 V, for obtaining high quality images of selected specimens.

### Histological and immunohistochemistry analysis

Each sample was independently reviewed by two blinded researchers. HE and MT staining were used to evaluate epidermolysis and necrosis. BD-2 and BD-3 expression was semi quantitatively assessed by a staining intensity score (0, negative; 1, weak; 2, moderate; 3, strong) in the skin and perivascular regions^59^. When differences were found, specimens were again reviewed by the two researchers until a consensus was reached.

### Quantification of bacteria and leucocytes on the surface of the foreign body by scanning electron microscopy

Bacteria, leucocytes and phagocytes were identified on the surface of catheters as described by van Gennip *et al*., Polliack and Saint-Guillain *et al*., respectively^60–62^. Only catheters collected from rats that had survived the entire experiment (7 days) were used for counting purposes. Average bacterial density on the surface of the catheter was based on manual counting bacteria in 20 random SEM fields at a 7500X magnification on each of the two catheter segments in each rat by a blinded researcher. When only one catheter segment could be retrieved, average bacterial density was done analogously using 40 random SEM fields at 7500X magnification. When more than 300 bacteria were found in a given SEM field, bacteria were considered uncountable. Using the same methodology, the disposition of bacteria in each SEM field was recorded as planktonic, biofilm or mixed.

Average leucocyte density on the surface of the catheter was performed in a similar fashion, with the exception that SEM fields used were obtained at 750X magnification. The average phagocyte density on the surface of catheters was determined in the same way. Phagocytes were defined as those leucocytes that showed evidence of pseudopods in direct contact with bacteria and/or biofilm.

Bacteria and leucocyte were included in counts only if the top upper edge of the cell was in the SEM field^63,64^. SEM fields with clots or largely occupied by biofilms were discarded, as bacteria were not accessible to counting using the methodology employed.

### Statistical analysis

Qualitative variables were expressed as percentages. Quantitative variables were expressed as means ± standard deviation. The SPSS 21.0 ® software was used for descriptive and inferential statistical analysis. The Kolmogorov-Smirnov test was used to assess if variables were normally distributed. ANOVA and t-Student test were used to compare averages in normally distributed data. Kruskal-Wallis and Mann-Whitney tests were used to compare means in non-normally distributed data. Wilcoxon rank-sum test was used to compare ordinal data. Proportions were analyzed with the Chi-square test or Fisher’s exact test. Association between numerical variables was assessed using Pearson’s correlation coefficient. Relationship between ordinal variables was evaluated with resort to Spearman Rank Correlation Coefficient^65,66^. Differences in survival in the different experimental groups was tested with the log rank test. A two-tailed p < 0.05 was considered to be statistically significant.

## Results

### Inoculation of arterialized venous flaps results in a model of persistent ischemic flap infection

In order to obtain a rat model of ischemia and *P*. *aeruginosa* infection in the presence of a foreign body, we followed the steps summarized in Fig. 2. Ischemia was obtained by using a variant of the rat abdominal arterialized venous flap, established by our group^42^. Infection was obtained by inoculating *P*. *aeruginosa* and two 1-cm segments of sterile 14-gauge silicone catheters were used to simulate foreign body.

Serial *P*. *aeruginosa* concentrations (10^3^; 10^4^; 10^5^; 10^6^ and 10^7^ CFU/ml) were tested in the mentioned model. A target concentration of 10^5^ CFU/ml was selected, since it caused no rat mortality within 7 days and systematically resulted in clinically significant infection and partial flap necrosis. Higher concentrations led to complete or nearly complete flap necrosis (10^6^ CFU/ml; n = 2) and animal’s death (10^7^ CFU/ml; n = 3), whereas lower concentrations (10^3^ and 10^4^ CFU/ml; n = 3 for each concentration) did not produce identifiable infection.

**Figure 3:**
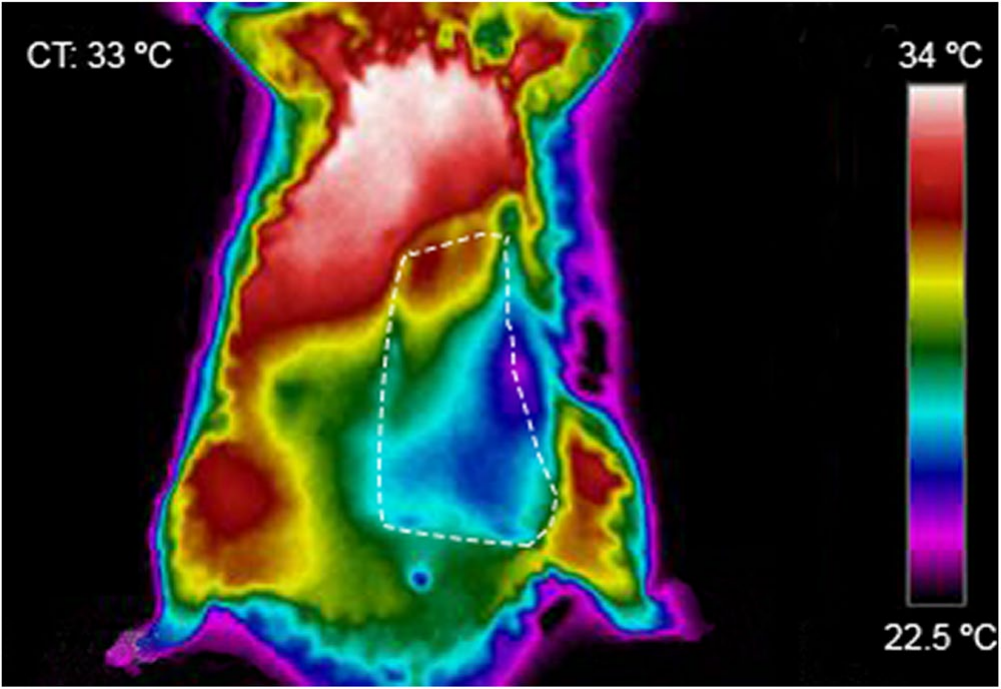
Representative direct infrared thermography image of the ventrolateral aspect of the abdomen of the rat 1 hour postoperatively. Flap boundaries are highlighted with the interrupted lines. This image illustrates that the temperature of the flap’s surface is inferior to the contralateral side, due to the flap’s relative ischemia, which results from its unconventional pattern of perfusion. CT, rat’s core temperature.

By thermography analysis, one hour after surgery, the average difference between the flap surface’s temperature and the temperature of the surface of the homologous contralateral region was 2.34 ± 1.06 °C (p < 0.001). Figure 3 shows the typical thermographic appearance of the flap one hour after surgery, illustrating the lower temperature of the flap compared to the contralateral side. Throughout the experiment, the flap’s temperature remained inferior to that of the homologous contralateral region.

Overall our data shows that a model of ischemia, infection and foreign body can be successfully obtained by adapting our previous established model of the rat abdominal arterialized venous flap, following inoculation with *P*. *aeruginosa* and introduction of silicone catheters.

### BD-2 and BD-3 increase flap survival rates

We then assessed whether overexpression of BDs in the flaps could improve survival and reduce infection. For this purpose, we performed a set of experiments in rats that included flaps’ transduction with lentiviruses containing the BD-2 and BD-3 genes, as represented in Figs 1 and 2.

**Figure 4:**
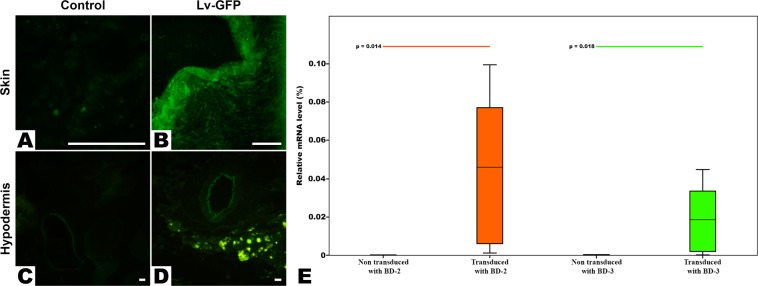
Confirmation of flap transduction. (**A** to **D**) Typical fluorescence image photographs of the skin and hypodermis of the flap of a rat transduced with a Green Fluorescent Protein coding lentivirus (LV-GFP) and those of the flap of a non-transduced rat (Control) seven days after surgery. These photographs demonstrate transduction of flaps by the virus. Calibration bar = 100 µm (**E**). Analysis of the BD-2 and BD-3 mRNA expression in rat flaps. Box plots represent the relative mRNA expression level of human β-defensins 2 and 3 (BD-2 and 3) analyzed in transduced flaps (BD-2, BD-3 and BD-2 + BD-3) compared to non-transduced flaps on the seventh post-operative day relatively to the expression of the β-actin in these flaps using real-time PCR. The relative mRNA levels of each gene are expressed as the percentage of the β-actin mRNA levels. Flaps transduced with BD-2 include the BD-2 and the BD-2 + BD-3 groups. Similarly, flaps transduced with BD-3 include the BD-3 and the BD-2 + BD-3 groups. Horizontal lines in the upper portion of the figure indicate statistically significant differences between groups (p < 0.05).

To confirm transduction efficiency, seven days after surgery, flaps from rats transduced with GFP were analyzed by fluorescence microscopy. As shown in Fig. 4, flaps from GFP lentivirus-transduced rats showed GFP expression. The expression was greater in endothelia and in the epidermis (Fig. 4). Quantification of BD-2 mRNA expression by real-time PCR seven days after transduction, revealed 591.12 × 10^6^% ± 894.00 × 10^6^% relative expression of BD-2 in transduced flaps versus 3.53 × 10^6^% ± 11.35 × 10^6^% relative expression in non-transduced flaps (p = 0.014) (Fig. 4). Analogously, the relative BD-3 expression determined in a similar fashion was 253.09 × 10^6^% ± 354.61 × 10^6^% in transduced flaps and 25.94 × 10^6^% ± 87.44 × 10^6^% for non-transduced flaps (p = 0.018) (Fig. 4). Using the same methodology, no statistically significant differences were found in the relative expression of BD-2 in the BD-2 and in the BD-2 + BD-3 groups, nor in the relative expression of BD-3 in the BD-3 and in the BD-2 + BD-3 groups.

**Figure 5:**
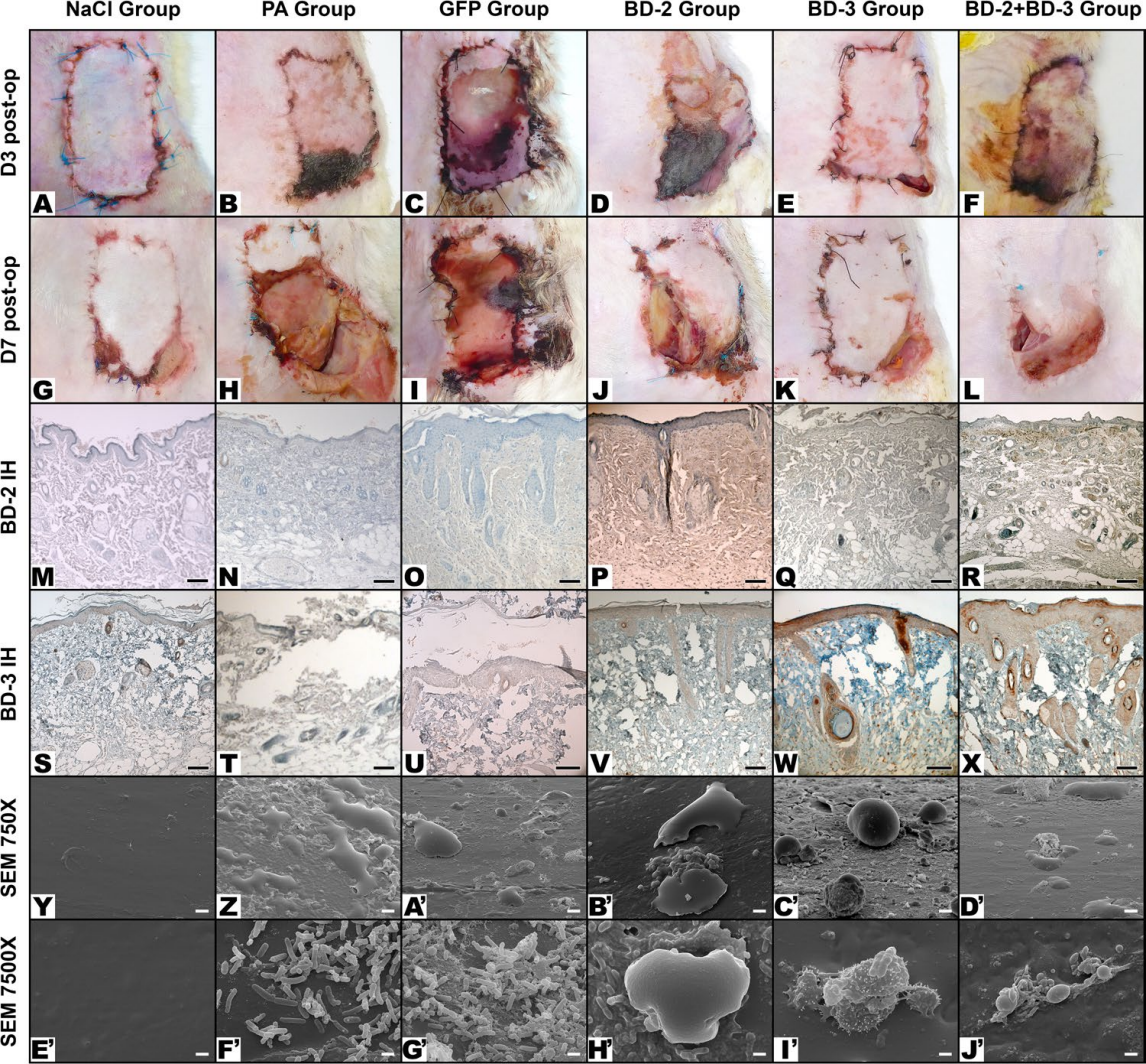
Typical macroscopic and microscopic features of flaps in the different experimental groups. Macroscopic images were taken on the third day post-operatively (D3 Post-op), and seven days post-operatively (D7 Post-op). Microscopic images were taken with immunohistochemical staining with anti-BD-2 (BD-2 IH), or anti-BD-3 (BD-3 IH) antibodies of flap biopsies performed on the seventh day after the surgery. Scanning electron microscopy images (SEM) of the surface of catheters were taken at 750X or 7500X amplification. Figures (**Y** to D’) show the typical aspect of leucocytes on the surface of foreign bodies. In figures (E’ to J’) the characteristic features of bacteria on the surface of foreign bodies using the magnifications employed for counting purposes are illustrated. Calibration bar = 100 µm (**M**,**N**, **P**–**T**, **V**–**X**); 25 µm (**O**, **U**); 10 µm (**Y**–D’); 1 µm (E’–J’).

**Figure 6:**
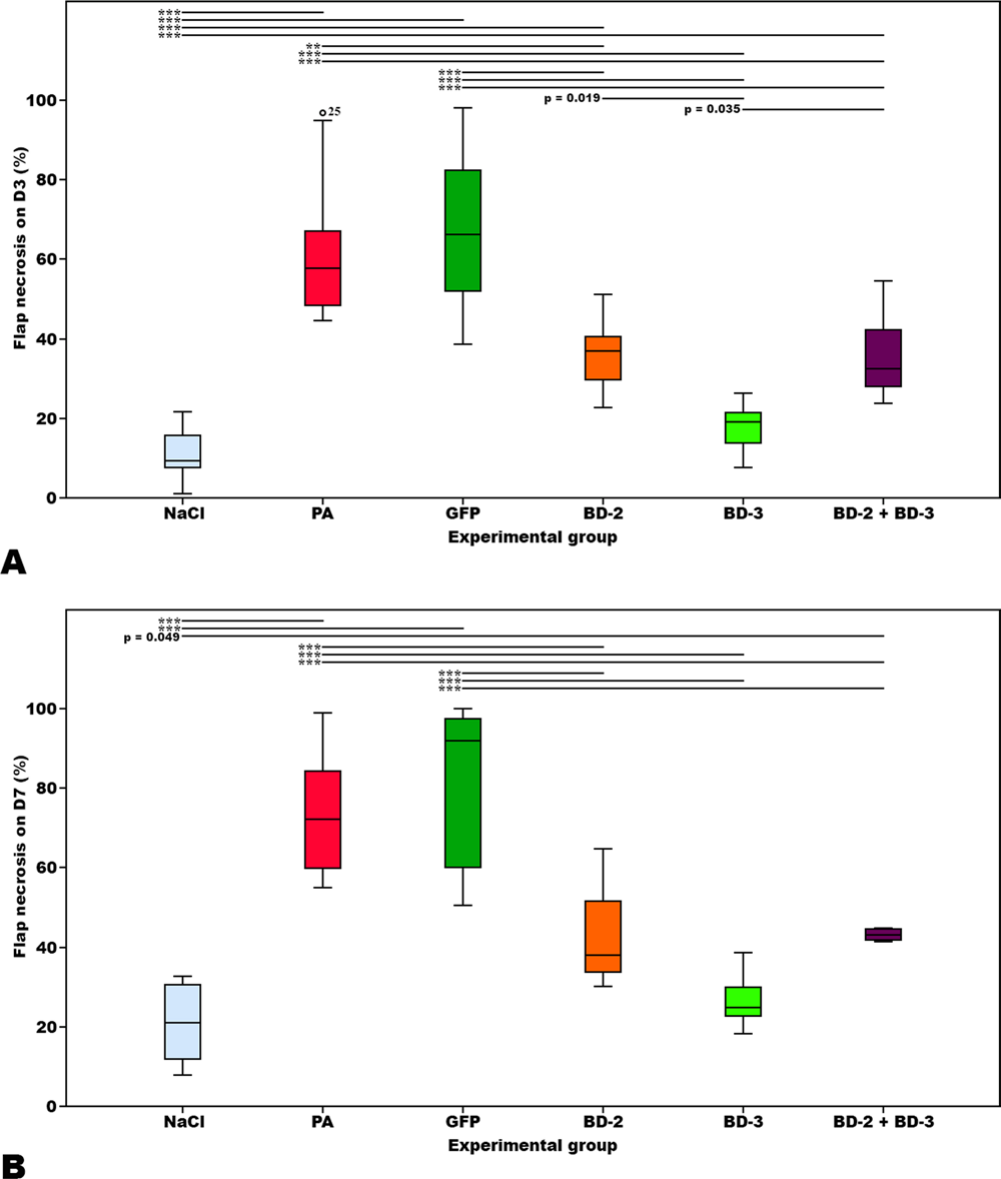
Bar graphs representing the percentage of flap necrosis relatively to the flap original surface area on the third (**A**) and seventh (**B**) postoperative days in the different experimental groups. Horizontal lines in the upper portion of the figure indicate statistically significant differences between groups (p < 0.05). Error bars indicate 95% confidence intervals. **p < 0.01. ***p < 0.001.

Figure 5 summarizes the main clinical, optical microscopy and SEM findings in the different experimental groups. On the third postoperative day, the average area of necrotic flap was higher in the *P*. *aeruginosa* (61.98% ± 18.52%) and GFP (67.39% ± 20.67%) groups than in the groups BD-2 (37.16% ± 9.04%), BD3 (17.79% ± 5.48%) and BD-2 + BD-3 (35.09% ± 9.43%) (p ≤ 0.001). The necrotic area was the smallest in the NaCl (11.07% ± 5.72%) and in the BD-3 groups. There was no statistically significant difference between these two groups (p = 0.836) (Fig. 6).

On the seventh postoperative day, lower average flap necrosis rates were also found in the NaCl (20.88% ± 9.94%), BD-2 (42.81% ± 13.56%), BD-3 (25.39% ± 8.50%) and BD-2 + BD-3 (49.97% ± 11.37%) groups than in the *P*. *aeruginosa* (74.30% ± 16.43%) and GFP (82.53% ± 19.73%) groups (p < 0.001). Although slightly higher, the average necrosis rates of groups BD-2 and BD3 was not significantly different from that of the NaCl group. However, the average necrosis rate of BD-2 + BD-3 was higher than that of the NaCl group (p = 0.049) (Fig. 6).

In summary, our results demonstrate that transduction of flaps with BD-2 or BD-3 improves infected flap survival at day 7 following surgery, by decreasing tissue necrosis.

### Evidence of flap ischemia and defensin expression by histology and immunohistochemistry

Histological analysis of the flaps revealed signs of venous congestion, marked edema, epidermolysis and areas of necrosis in all experimental groups. However, these changes were more pronounced in the *P*. *aeruginosa* and GFP groups (Fig. 5). Immunohistochemistry confirmed expression of BD-2 and BD-3 in the transduced rats (Fig. 5). BD-2 expression occurred mainly in endothelia and perivascular tissues, although it was also observed in fibroblasts and in the epidermis. BD-3 expression was strongest in the epidermis and skin appendages (Fig. 5). Using the mentioned semi-quantitative score for immunohistochemistry staining^59^, BD-2 expression was as follows: 0.25 ± 0.45 for the NaCl group; 0.23 ± 0.44 for the PA group; 0.36 ± 0.50 for the GFP group; 2.07 ± 0.59 for the BD-2 group; 0.13 ± 0.35 for the BD-3 group; 1.64 ± 0.50 for the BD2 + BD-3 group. Proceeding in a similar way, BD-3 expression in the different experimental groups was the following: 0.67 ± 0.49 for the NaCl group; 0.69 ± 0.48 for the PA group; 0.79 ± 0.70 for the GFP group; 0.27 ± 0.59 for the BD-2 group; 2.80 ± 0.41 for the BD-3 group; 2.50 ± 0.52 for the BD2 + BD-3 group. BD-3 expression was stronger than BD-2 expression. However, this difference did not reach statistically significance.

### BD-2 and BD-3 decrease bacterial numbers and biofilms on the surface of foreign bodies

We then assessed the number of bacteria on the surface of the foreign body in the different experimental groups. At the end of the experiment, it was possible to retrieve 56 catheters that were distributed as follows: 8 in the NaCl group, 6 in the PA group, 12 in the GFP group, 9 in the BD-2 group, 11 in the BD-3 group, and 10 in the BD-2 + BD-3 group.

In all rats, infection was confirmed by growth in *P*. *aeruginosa* MacConkey agar, Gram stain and positive oxidase reaction. Table 1 summarizes bacterial counts determined by different methods on the 7^th^ day postoperatively in the different experimental groups.

**Table 1:** Average values of bacterial counts determined by different methods on the 7^th^ day postoperatively in the different experimental groups. For each rat, the average number of bacteria on the surface of catheters was based on manual counting bacterial cells on 20 SEM fields at 7500X magnification on each of the two catheter segments, or, when only one catheter segment could be retrieved, on counts performed on 40 SEM fields of that catheter segment. A scanning electron microscope JEOL JSM-5410, with acceleration voltage of 0.015-0.030 V, was used for quantification purposes. Values are expressed as average ± standard deviation. CFU, colony forming units; SEM, Scanning electron microscopy.

Experimental Group	Viable bacteria cultured from skin flap biopsy (CFU/mg)	Real-time PCR from skin flap biopsy (ng/µl)	Bacteria on the surface of the foreign body (n/SEM field)
NaCl	2.42 × 10^5^ ± 5.87 × 10^5^	1.23 × 10^−5^ ± 2.06 × 10^−5^	7.74 ± 8.18
PA	6.98 × 10^6^ ± 1.17 × 10^7^	1.26 × 10^−1^ ± 1.17 × 10^7^	71.70 ± 47.4
GFP	9.46 × 10^5^ ± 6.46 × 10^5^	7.22 × 10^0^ ± 1.36 × 10^1^	92.75 ± 35.9
BD-2	8.06 × 10^5^ ± 8.59 × 10^5^	5.52 × 10^−1^ ± 7.97 × 10^−1^	67.08 ± 42.4
BD-3	2.51 × 10^5^ ± 5.88 × 10^5^	1.13 × 10^−5^ ± 1.39 × 10^−5^	19.37 ± 16.3
BD-2 + BD-3	3.55 × 10^5^ ± 4.52 × 10^5^	1.94 × 10^−3^ ± 2.92 × 10^−3^	54.04 ± 41.6
Statistical analysis summary	No significant differences were found	No significant differences were found	NaCl < PA; p = 0.013
			NaCl < GFP; p < 0.001
			NaCl < BD-2; p = 0.010
			PA > BD-3; p = 0.042
			GFP > BD-3; p < 0.001
			BD-2 > BD-3; p = 0.035

For each rat, the average number of bacteria on the surface of catheters was based on manual counting bacterial cells on 20 SEM fields at 7500X magnification on each of the two catheter segments, or, when only one catheter segment could be retrieved, on counts performed on 40 SEM fields of that catheter segment. A scanning electron microscope JEOL JSM-5410, with acceleration voltage of 0.015–0.030 V, was used for quantification purposes.

Values are expressed as average ± standard deviation.

CFU, colony forming units; SEM, Scanning electron microscopy

**Figure 7:**
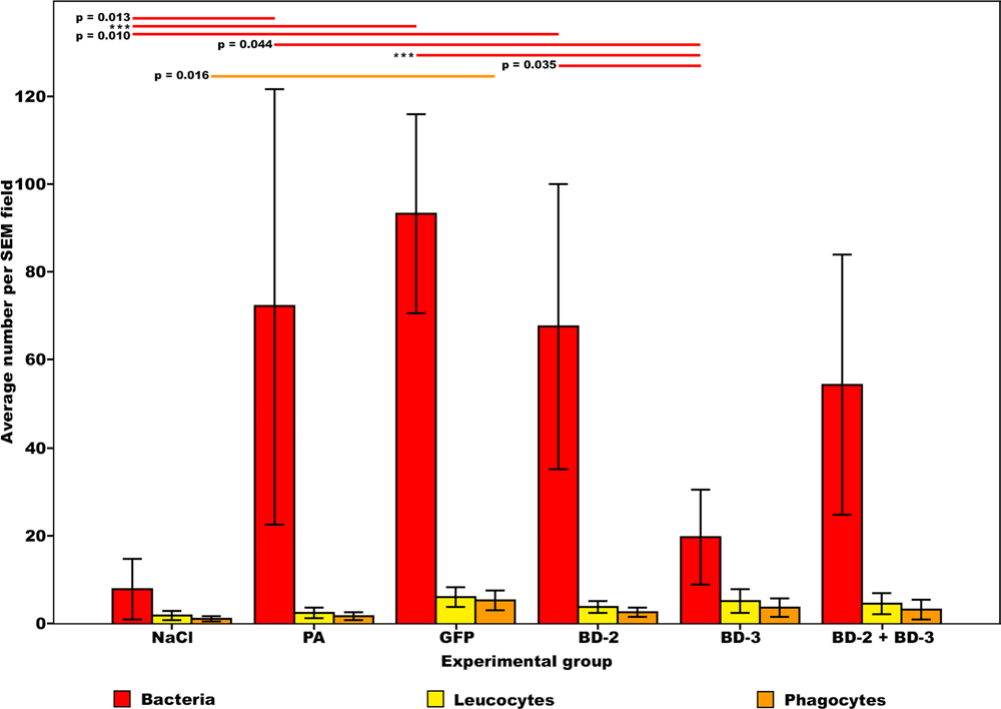
Bar graphs representing the average number of bacteria, leucocytes and phagocytes on the surface of the catheter segments placed underneath the flaps per scanning electron microscopy (SEM) field. For each rat, the average number of bacteria on the surface of catheters was based on manual counting bacterial cells on 20 SEM fields at 7500X magnification on each of the two catheter segments, or, when only one catheter segment could be retrieved, on counts performed on 40 SEM fields of that catheter segment. Average leucocyte and phagocyte density on the surface of the catheter was performed in a similar way, with the exception that SEM fields used were at obtained 750X magnification. A scanning electron microscope JEOL JSM-5410, with acceleration voltage of 0.015–0.030 V, was used for quantification purposes. Horizontal lines in the upper portion of the figure indicate statistically significant differences between groups (p < 0.05). Error bars indicate 95% confidence intervals. ***p < 0.001.

As shown in Table 1, significant differences were found in the number of bacteria on the surface of the foreign body (Fig. 7). The number of bacteria on the surface of catheters was lower in the NaCl, BD-3, and BD-2 + BD-3 groups. There were no statistically significant differences between these 3 groups. The BD-3 group presented a smaller number of bacteria on the surface of catheters than the *P*. *aeruginosa* (p < 0.042), GFP (p < 0.001) and BD-2 (p = 0.035) groups.

**Figure 8:**
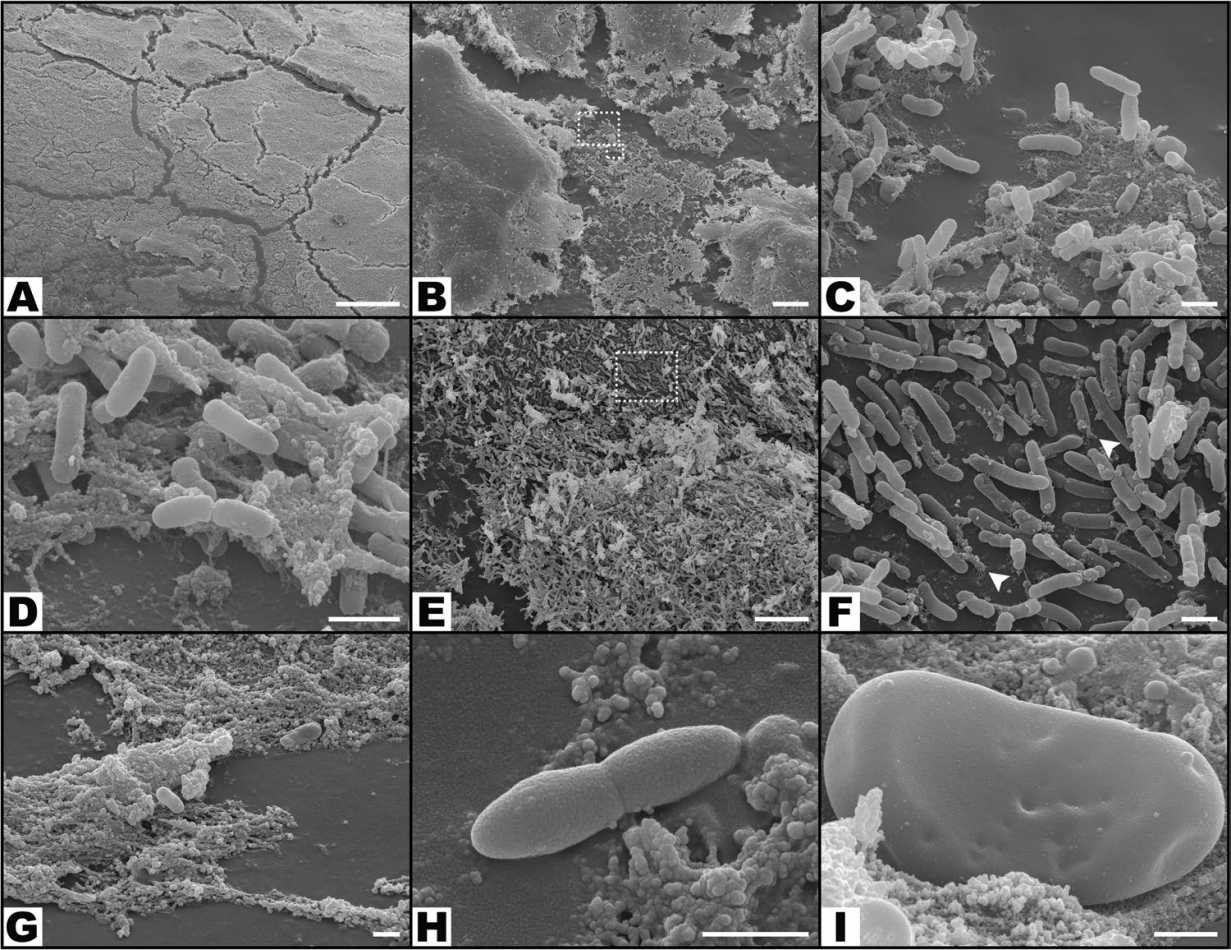
Morphological features of bacteria on the surface of the foreign body in increasing magnifications by scanning electron microscopy. (**A**,**B**) Flat biofilm on the surface of a catheter segment. (**C**) Magnification of the large rectangular area in the center of (**B**) showing *Pseudomonas aeruginosa* and associated biofilm. (**D**) Magnification of the small rectangular area in the center of (**B**) showing *Pseudomonas aeruginosa* bacterial cells dividing in the biofilm. (**E**) Mushroom-shaped biofilm with uncountable bacterial cells. (**F**) Higher magnification view of the rectangular dotted area in the center of (**E**) showing bacterial division and adherence to the surface of the catheter (arrow heads). (**G**) Biofilm covering most of *P*. *aeruginosa* cells. (**H**) High magnification view of a *P*. *aeruginosa* cell dividing on the surface of the catheter. (**I**) High magnification image of a single *P*. *aeruginosa* cell on the surface of the biofilm showing the irregularities of the bacterial wall surface. Calibration bar = 100 µm (**A**) 10 µm (**B**,**E**) 1 µm (**C**,**D**,**F**,**I**).

**Figure 9:**
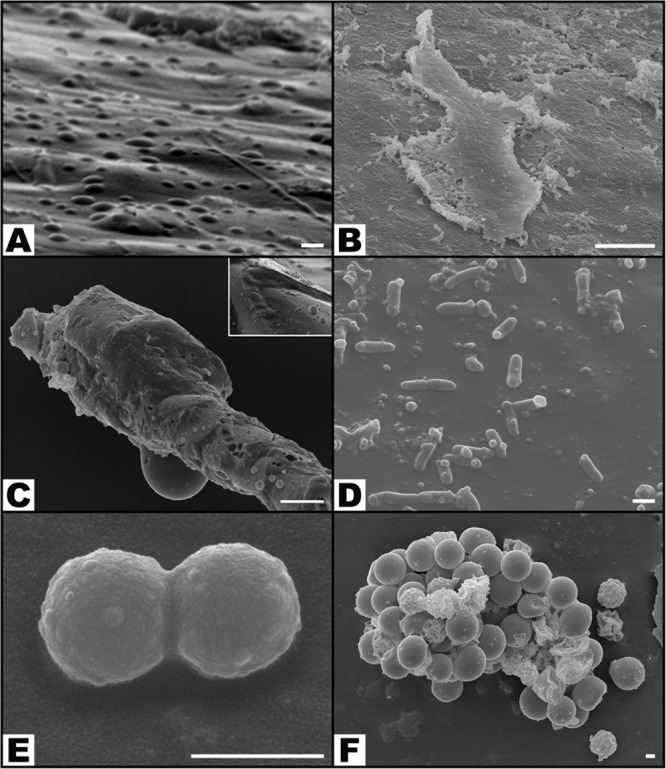
Typical scanning electron microscopy images of the surface of catheters showing the variable distribution of bacteria. (**A**) *Pseudomonas aeruginosa* in the planktonic form; (**B**) *P*. *aeruginosa* are seen forming a large flat biofilm in the central portion of the image; (**C**) Small *cocci* (contamination) are seen on the surface of an hair shaft that contaminated the surgical wound; the box represents an higher amplification view of the middle portion of the hair shaft (**D**) *P*. *aeruginosa* cells are seen scattered on the surface of the catheter; some of these cells are dividing; amongst *P*. *aeruginosa*, it is possible to observe cocci; (**E**) Diplococcus; (**F**) Staphylococcus. Calibration bar = 1 µm (A, D, E, F); 10 µm (**B**,**C**)

**Figure 10:**
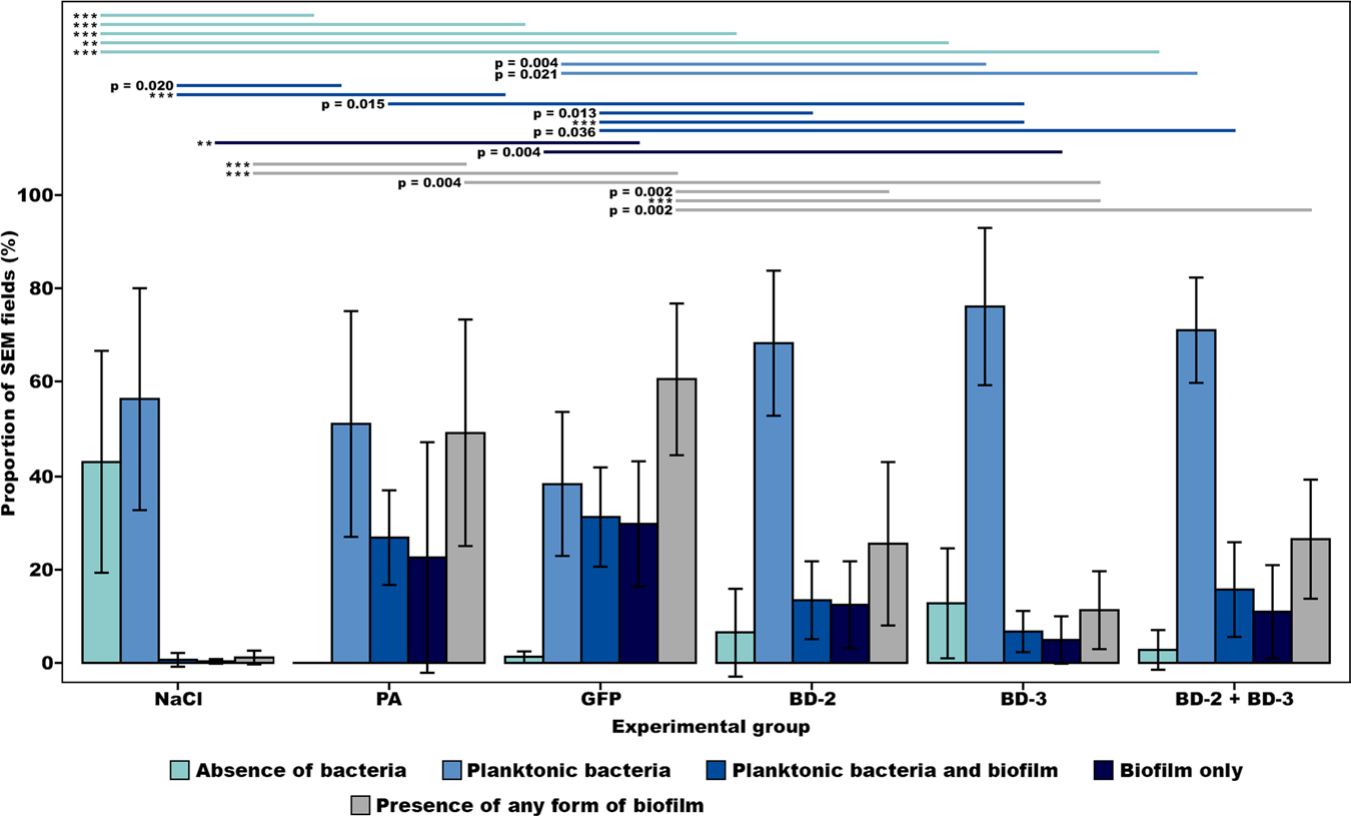
Bar graphs representing the proportion of scanning electron microscopy (SEM) fields in the different experimental groups with no bacteria, with planktonic bacteria, with both planktonic bacteria and biofilm, and only with biofilm. For each rat, observations were made on 20 random SEM fields at 7500X magnification on each of the two catheter segments, or, when only one catheter segment could be retrieved, on 40 random SEM fields of that catheter segment. Horizontal lines in the upper portion of the figure indicate statistically significant differences between groups (p < 0.05). Error bars indicate 95% confidence intervals. **p < 0.01, ***p < 0.001.

Concerning the distribution of bacteria on the surface of catheters (Figs 5E to J’, 8 to 10), the NaCl group presented the largest percentage of SEM fields without bacteria (42.81% ± 28.36%; p < 0.001), followed by the BD-3 group (12.72% ± 17.87%), the BD-2 group (6.39% ± 12.32%), and the BD-2 + BD-3 group (2.75% ± 6.17%) (Fig. 10). The percentage of SEM fields without evidence of biofilm formation were also more numerous in the NaCl (99.06% ± 1.86%), BD-2 (74.44% ± 22.63%), BD-3 (88.59% ± 12.40%), and BD-2 + BD-3 (73.50% ± 17.72%) groups compared to the control groups *P*. *aeruginosa* (50.83% ± 23.06%) and GFP (39.38% ± 25.30%). This difference in biofilm presence of the former four groups compared to the GFP control group was statistically very significant (p ≤ 0.002) (Fig. 10).

**Figure 11:**
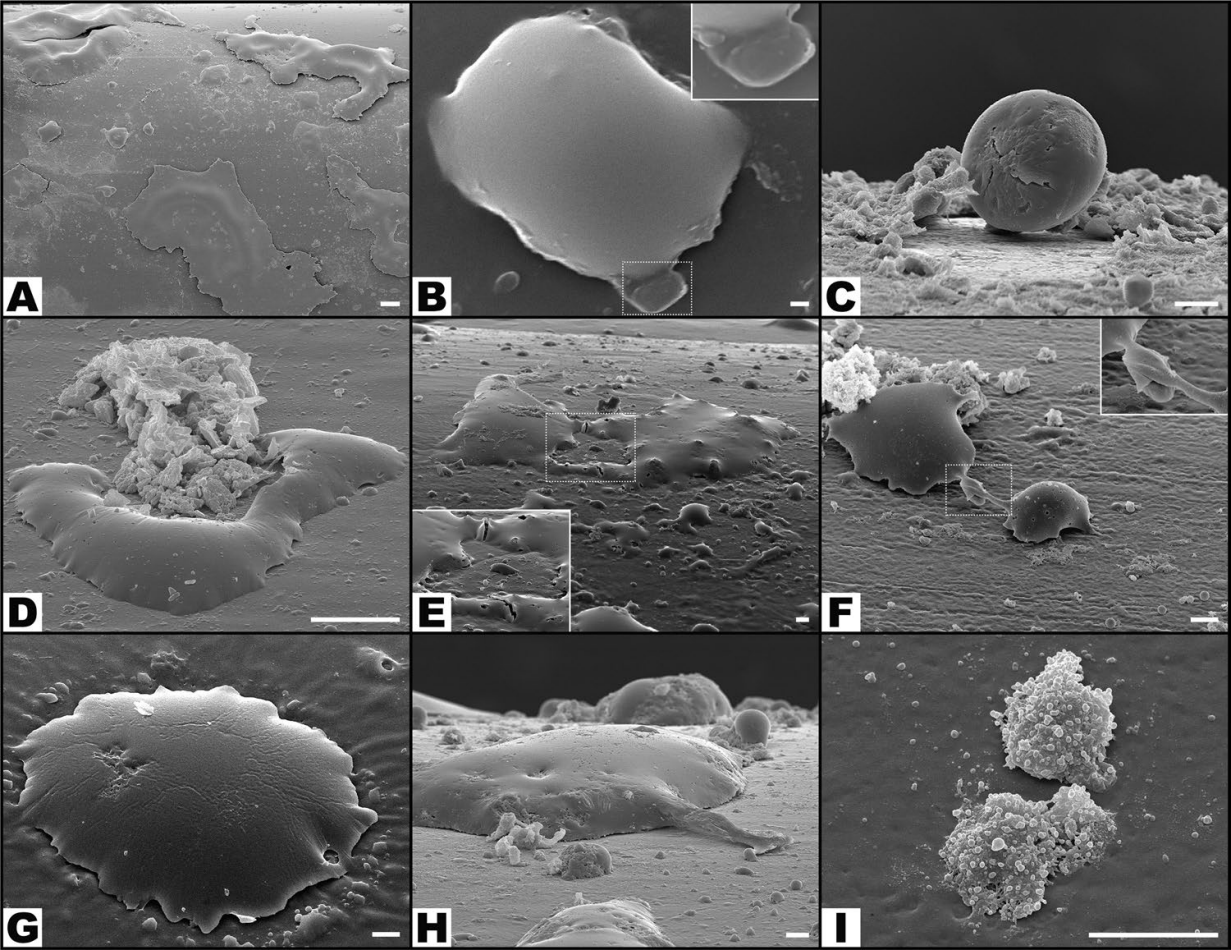
Typical scanning electron microscopy images of the surface of catheters showing multiple features of leucocyte morphology and interaction with the surrounding environment. (**A**) Low magnification view of the surface of the catheter showing giant leucocytes interspersed with smaller leucocytes. (**B**) Leucocyte engulfing a *Pseudomonas aeruginosa* cell in the area highlighted with the interrupted line box; on the top right corner of the picture there is a higher amplification view of this interaction. (**C**) Leucocyte adhering to the catheter’s surface. (**D**) A leucocyte phagocytosing a region with biofilm. (**E**,**F**) leucocytes interacting on the surface of the catheter (dotted boxes highlight amplified views of these interactions). (**G**) Large leucocyte engulfing adjacent biofilm. (**H**) In the central portion of the image there is a large leucocyte extending a pseudopod into adjacent *P*. *aeruginosa*. (**I**) Three leucocytes with multiple vesicles on their surface. Calibration bar = 100 µm (**A**) 1 µm (**B**) 10 µm (**C** to **I**).

Numerous leucocytes could be observed on the surface of catheters in all groups. (Figs 5Y to D’, 7 and 11). Apart from the average number of leucocytes being superior in the GFP group (4.92 ± 3.68 leucocytes/SEM field) than in the NaCl group (0.75 ± 0.93 leucocytes/SEM field; p = 0.016), no other statistically significant differences were found in leucocytes’ and phagocytes’ numbers on the surface of catheters in the remaining experimental groups (Fig. 7).

Bacterial contamination with cocci were observed in 8.92% ± 22.21% of SEM fields (Fig. 9). There were no statistically significant differences between the different experimental groups.

Overall, data shows that in the model used the surface of foreign bodies presents significant numbers of bacteria. Flap transduction with either BD-2 or BD-3, and in particular with the latter, remarkably lowers bacterial numbers on the surface of foreign bodies.

### Bacterial counts and biofilms on the surface of the foreign body were correlated with flap necrosis

We then assessed whether bacteria accumulation on the surface of the foreign body was correlated with flap necrosis. Table 2 synthetizes the most relevant correlations in the data obtained in this study.

**Table 2:** Synthesis of the most relevant correlations in the data obtained in this study.

Variable	Variable	Pearson’s correlation factor	P value
Flap necrosis rate on the 3^rd^ postoperative dayFlap necrosis rate on the 7^th^ postoperative day	Flap necrosis rate on the 7^th^ day	0.922	p < 0.001
	Average number of bacteria per SEM field	0.609	p < 0.001
	Percentage of SEM fields with biofilm	0.596	p < 0.001
	Percentage of SEM fields with planktonic bacteria	−0.409	p = 0.004
	Percentage of SEM fields without bacteria	−0.426	p = 0.003
	Percentage of SEM fields without biofilm	−0.681	p < 0.001
	*P*. *aeruginosa* counts on the 3^rd^ postoperative day determined by culture of flap biopsies	0.287	p = 0.041
	Average number of bacteria per SEM field	0.626	p < 0.001
	Percentage of SEM fields with biofilm	0.563	p = 0.001
	Percentage of SEM fields with planktonic bacteria	−0.379	p = 0.03
	Percentage of SEM fields without bacteria	−0.462	p = 0.007
	Percentage of SEM fields without biofilm	−0.674	p < 0.001
	*P*. *aeruginosa* counts on the 3^rd^ postoperative day determined by culture of flap biopsies	0.395	p = 0.016
Average number of bacteria per SEM field	Average number of leucocytes per SEM field	0.276	p = 0.041
	Average number of phagocytes per SEM field	0.401	p = 0.002

SEM, scanning electron microscopy.

Flap necrosis rates on the 3^rd^ and 7^th^ day postoperatively were highly correlated (Pearson’s correlation factor = 0.922; p < 0.001). Flap necrosis was also correlated with several features of catheters’ surface. In fact, flap necrosis rate on the third postoperative day was positively correlated with the average number of bacteria per SEM field and the percentage of SEM fields with biofilm (Pearson’s correlation factor = 0.609; p < 0.001 and 0.596; p < 0.001, respectively). It was negatively correlated with the following variables: percentage of SEM fields with planktonic bacteria; percentage of SEM fields without bacteria; percentage of SEM fields without biofilm (Pearson’s correlation factor = - 0.409, p = 0.004; - 0.426, p = 0.003 and - 0.681; p < 0.001, respectively).

Analogously, flap necrosis on the seventh day postoperatively was positively correlated with the following findings: average number of bacteria per SEM field (Pearson’s correlation factor = 0.626; p < 0.001) and the percentage of SEM fields with biofilm (Pearson’s correlation factor = 0.563; p = 0.001). Moreover, flap necrosis rate on the seventh day after surgery was significantly negatively correlated with the percentage of SEM fields with planktonic bacteria, without bacteria and without biofilm (Pearson’s correlation factor = - 0.379, p = 0.03; - 0.462, p = 0.007; - 0.674, p < 0.001, respectively).

The average number of bacteria per SEM field was also positively correlated with the number of leucocytes (Pearson’s correlation factor = 0.276; p = 0.041); and phagocytes per SEM field (Pearson’s correlation factor = 0.401; p = 0.002).

**Figure 12:**
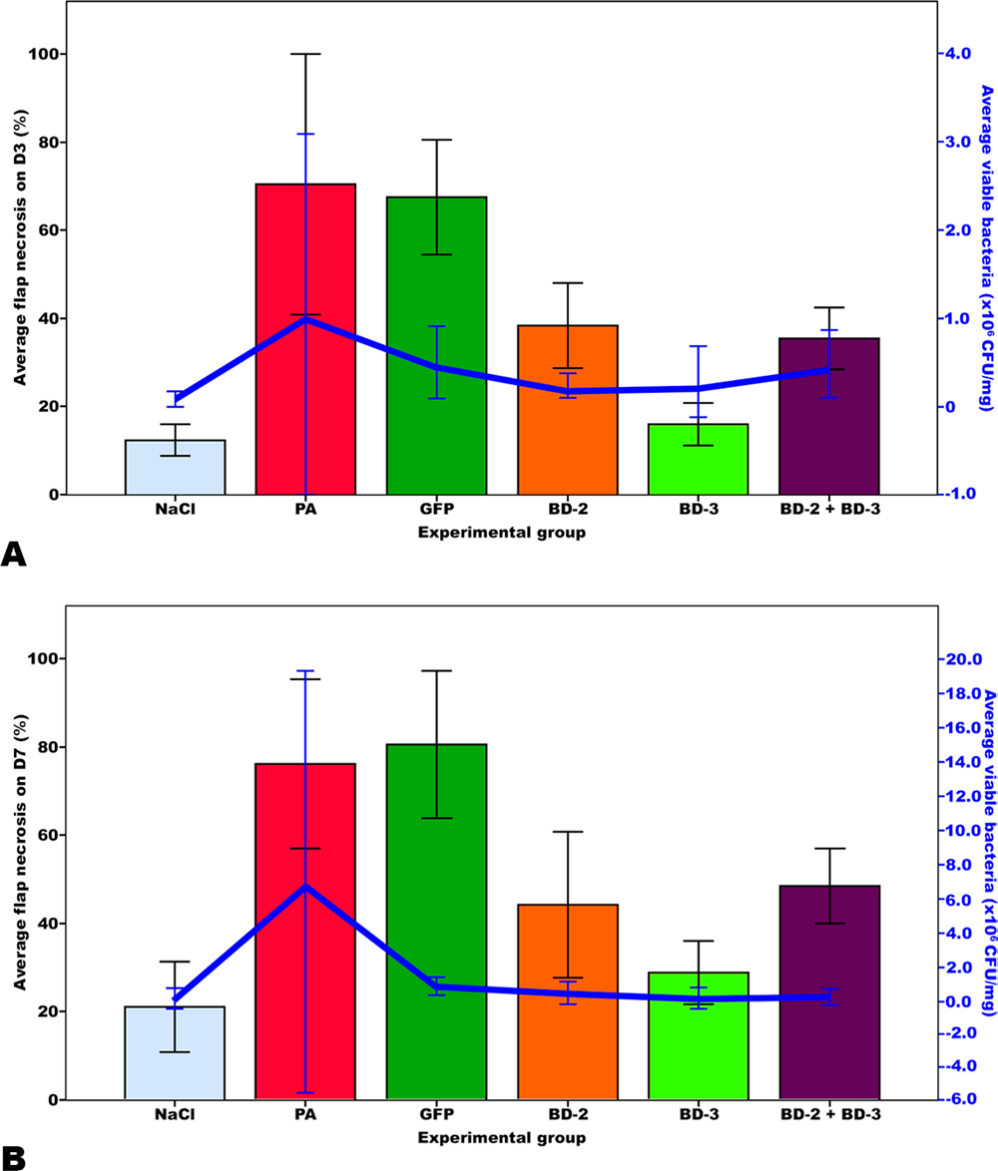
Graphic representation of the relation between the proportion of flap necrosis (expressed as percentage of flap’s initial area) and bacterial counts after flap biopsy on the third (**A**) and seventh (**B**) days after surgery in the different experimental groups. On the both days, flap necrosis and bacterial counts were lower in the animals expressing human β-defensins 2 and 3. Error bars indicate 95% confidence intervals.

*P*. *aeruginosa* counts on the 3^rd^ postoperative day determined by culture of flap biopsies, were correlated with flap necrosis on that day (Pearson’s correlation factor = 0.287; p = 0.041) and on the seventh day after surgery (Pearson’s correlation factor = 0.395; p = 0.016) (Fig. 12). However, there were no statistically significant correlations between *P*. *aeruginosa* counts after flap biopsy on the seventh day after surgery and flap necrosis either on the 3^rd^ or 7^th^ postoperative days. (Fig. 12).

There were no statistically significant correlations between bacterial quantification after flap biopsy and culture or real-time PCR, on the one hand, and SEM bacterial counts on the other hand.

In conclusion, our data shows that the bacterial counts on the surface of the foreign body and biofilm formation were correlated with flap necrosis.

## Discussion

Our study showed that transducing an ischemic skin flap in the rat with human β-defensins, the BD-2 and BD-3 genes, increased skin flap survival in the context of a *P*. *aeruginosa* infection in the presence of a foreign body. In addition, BD-2 and particularly BD-3 transduction reduced *P*. *aeruginosa* numbers and biofilm formation on the surface of the foreign body. Other authors had already shown that BD-2 and BD-3 are overexpressed in the presence of *P*. *aeruginosa* keratitis in mice and that overexpression was associated with diminished *P*. *aeruginosa* numbers in the eyes of affected mice^15,67^.

However, as far as the authors could determine, this is the first description of the transduction of BDs genes to treat a *P*. *aeruginosa* infection *in vivo*^14^. It is also the first time that the transduction of an AMP gene is performed with the intent to treat an infection taking place in a poorly perfused region, simulating local ischemia. This scenario is frequent in the clinical setting, occurring after trauma, radiotherapy or in ischemic regions of the body, namely in the lower limbs of atherosclerotic and/or diabetic patients^68,69^. This study describes for the first time the morphometric features of the bacteria on the surface of a foreign body and thoroughly compares them with the clinical evaluation of the overlying skin flap, and with bacterial quantification using microbiological cultures of skin biopsy and real-time PCR. Other authors had already shown the possibility of treating a *Staphylococcus aureus* infection transducing a normally perfused flap with the AMP cathelicidin LL-37^43^.

The groups treated with BDs presented a smaller percentage of SEM fields with any form of biofilm relatively to the GFP group (p ≤ 0.002; Fig. 10). Arguably, the GFP group seems to be the most appropriate control group, as it involves expression of a foreign protein using a viral vector. This alone has been shown to affect local bacterial clearance^70^. These data suggest that *in vivo* these AMPs are able to prevent the formation of biofilms, which are one of the most common causes of clinical persistent infection^71–76^. This adds to the knowledge of BD physiology, as most papers in this field refer to the action of AMPs *in vitro*, not addressing the role of these substances *in vivo*^77^.

Flap necrosis rates on the 3^rd^ and 7^th^ day postoperatively were highly correlated (Pearson’s correlation factor = 0.922; p < 0.001). This suggests that, in this rodent model, the ongoing *P*. *aeruginosa* infection associated with the foreign body led to continuous breakdown of the overlying integument^72^. This had already been amply observed in clinical practice^72,78^. Recently, it has been shown that *P*. *aeruginosa* induces the production and release of cytotoxic amyloids with prion features^79,80^. It would be interesting to investigate in future studies whether this mechanism plays a significant role in the model used in the present work, and whether these processes could be curtailed or even halted by BDs transduction.

Interestingly, in this work, flap necrosis was significantly correlated with several features of catheters’ surface, namely bacterial numbers and biofilm presence. In opposition, in the present study, flap necrosis rates in the different groups was not correlated with bacterial determination by real-time PCR of flap biopsies. Moreover, only bacterial counts of microbiological cultures of biopsies of the flap performed on the third post-operative day correlated, and even then, only poorly, with flap necrosis rates on the third (Pearson’s correlation factor = 0.287; p = 0.041) and seventh (Pearson’s correlation factor = 0.395; p = 0.016) days after surgery. This lends support to the notion that in bacteria capable of producing biofilms, such as *P*. *aeruginosa*, the protracted release of bacterial cells from the biofilm medium is one of the main determinants of continuous tissue damage to the overlying tissues^45,60,72–74^. In fact, a recent meta-analysis demonstrated that biofilms are present in more than three quarters of chronic wounds, increasing their extension or preventing their closure^73^.

All these data suggest that the model herein used adequately mimics clinically relevant skin ulcers and infections. Noteworthy, a similar model had already been described by Van Wijngaerden *et al*., who introduced a catheter in the back of the rat to test antibiotic efficacy^45^. Furthermore, other authors, have also placed a catheter under a well perfused flap in the groin region of the rat^43^. Notwithstanding, these authors did not use SEM to evaluate the surface of the catheter used as a foreign body. It was only recently that van Gennip *et al*. emphasized the potential advantages of studying the morphology and interaction of bacteria and immune cells on the surface of foreign bodies with SEM^60^. However, these latter authors placed the foreign body inside the peritoneal cavity^60^.

We believe that our model presents the additional advantage of simulating an ischemic environment surrounding the foreign body. In fact, as shown in Fig. 5, one hour after surgery, the average temperature difference between the flap and the contralateral side was 2.34 ± 1.06 °C (p < 0.001). These data confirm that the surgical skin flap placed over the foreign body was ischemic, since skin temperature is known to be proportional to integumentary perfusion^81^.

In all groups, a 100 µl solution was injected intravascularly. To ensure that the same number of viral particles was administered in each of the transfected groups, in the BD-2 + BD-3 group, the solution was composed of 50 µl of the solution used in the BD-2 group and 50 µl of the solution used in the BD-3 group. The BD-2 + BD-3 group did not show statistically significant differences relatively to either the BD- 2 or the BD-3 groups, regarding flap necrosis on 7^th^ post-operative day (Fig. 6), in the number of bacteria (Fig. 7) or bacterial distribution on the surface of the foreign body (Fig. 10). Hence, these data suggest that the simultaneous expression of the two defensins does not have a synergistic or an antagonistic effect^82^.

One of the limitations of the present methodology was the impossibility to precisely differentiate between leucocytes types (Fig. 11)^60,83–87^. Although immunological methods have been proposed to distinguish leucocyte types using SEM, it has been shown that these methods may significantly affect leucocyte adhesion and morphology, making interpretation of data difficult^88^. In future works, it would be interesting to characterize with other techniques leucocyte numbers and cell types both on the surface of the foreign body and in the surrounding tissues.

Another limitation of this study was the presence of contamination of the wound by bacteria other than *P*. *aeruginosa*, namely by cocci, in 8.92 ± 22.21% of SEM fields (Fig. 9). This contamination affected all experimental groups equally. It is no doubt related to the ischemic nature of the skin flap used, leading to partial flap necrosis, surgical wound dehiscence and contamination with saprophytic flora. Hence, studies similar to this one but with normally perfused flaps are, therefore, warranted, in order to minimize contamination with other bacterial species.

The authors must concede that multiple other delivery methods of the BDs genes could have been employed^89,90^. Many of the other available vectors have potential advantages and disadvantages. For example, it has been shown that intradermal delivery of the transgenes might be superior to intravascular perfusion using lentivirus vectors in skin flaps^91^. Consequently, further studies are also necessary in this field, namely to test the superiority of other vectors, as well as the susceptibility of other bacteria species, particularly bacteria resistant to conventional antibiotics. Furthermore, it would be interesting to assess if BDs could be used to reverse established biofilm on the surface of foreign bodies.

Although the potential clinical merits of AMPs have been extoled for the past two decades, their clinical application has been tardy and incipient, due to potential systemic toxicity, susceptibility to proteases, and high cost of peptide production^10,11,13,14,92,93^. However, despite several *in vitro* studies (Supplemental Figs 1 and 2) many questions remain to be answered regarding the *in vivo* mechanisms of BD-2 and BD-3 bacterial clearance, biofilm impediment and increased tissue survival. The present work provides little help in answering these questions, making further studies warranted^4,14,20,94^.

Finally, the authors would like to note that controlled local production of a specific AMP by a flap placed over a difficult wound (for example, osteomyelitis in a mangled extremity) may theoretically allow to eradicate a bacterial infection and thus permit wound closure^43^. If the introduction of the AMP sequence was to cause oncogenesis, the flap could be simply removed and replaced by another flap or eventually by a skin graft^43,95^.

## Supplementary information

Supplementary Information
