# Supplementary material for: BD-2 and BD-3 increase skin flap survival in a model of ischemia and Pseudomonas aeruginosa infection
Source: Sci Rep. 2019 May 27;9:7854. doi: 10.1038/s41598-019-44153-y (PMC6536547; doi:10.1038/s41598-019-44153-y)
Supplement: Supplementary file 1 — Supplementary Information [file 41598_2019_44153_MOESM1_ESM.docx]

Manuscript title: BD-2 and BD-3 increase skin flap survival in a model of ischemia and *Pseudomonas aeruginosa* infection

**Short title:** BD-2 and BD-3 increase skin flap survival in a rat model of infection

**Authors:**

Diogo Casal^1-4 ¶ *^, Inês Iria^3,4,#a,#b ¶^, José S. Ramalho^4^, Sara Alves^5^, Eduarda Mota-Silva^6^, Luís Mascarenhas-Lemos^1,5^, Carlos Pontinha^1,5^, Maria Guadalupe-Cabral^4^, José Ferreira-Silva^5^, Mário Ferraz-Oliveira^5^, Valentina Vassilenko^6^, João Goyri-O’Neill^1^, Diogo Pais^1^, Paula A. Videira^3,4,7^

**Affiliations:**

1- Anatomy Department, NOVA Medical School, Universidade NOVA de Lisboa, Lisbon, Portugal

2- Plastic and Reconstructive Surgery Department and Burn Unit, Centro Hospitalar de Lisboa Central – Hospital de São José, Lisbon, Portugal

3- UCIBIO, Departamento de Ciências da Vida, Faculdade de Ciências e Tecnologia, Universidade NOVA de Lisboa, Caparica, Portugal

4- CEDOC, NOVA Medical School, Faculdade de Ciências Médicas, Universidade NOVA de Lisboa, Lisbon, Portugal

5- Pathology Department, Centro Hospitalar de Lisboa Central – Hospital de São José, Lisbon, Portugal

6- LIBPhys, Physics Department, Faculdade de Ciências e Tecnologias, Universidade NOVA de Lisboa, Caparica, Portugal

7- CDG & Allies- Professional and Patient Association International Network (PPAIN), Caparica, Portugal

#a- Molecular Microbiology and Biotechnology Unit, iMed, ULisboa, Faculty of Pharmacy, Universidade de Lisboa, Lisbon, Portugal

#b- INESC MN – Microsystems and Nanotechnologies, Instituto Superior Técnico, Universidade de Lisboa, Lisbon, Portugal

* Corresponding authors

E-mail: [diogo_bogalhao@yahoo.co.uk](mailto:diogo_bogalhao@yahoo.co.uk) (DC)

[p.videira@fct.unl.pt](mailto:p.videira@fct.unl.pt) (PAV)

¶ These authors contributed equally to this work.

**Supplementary Material:**

**
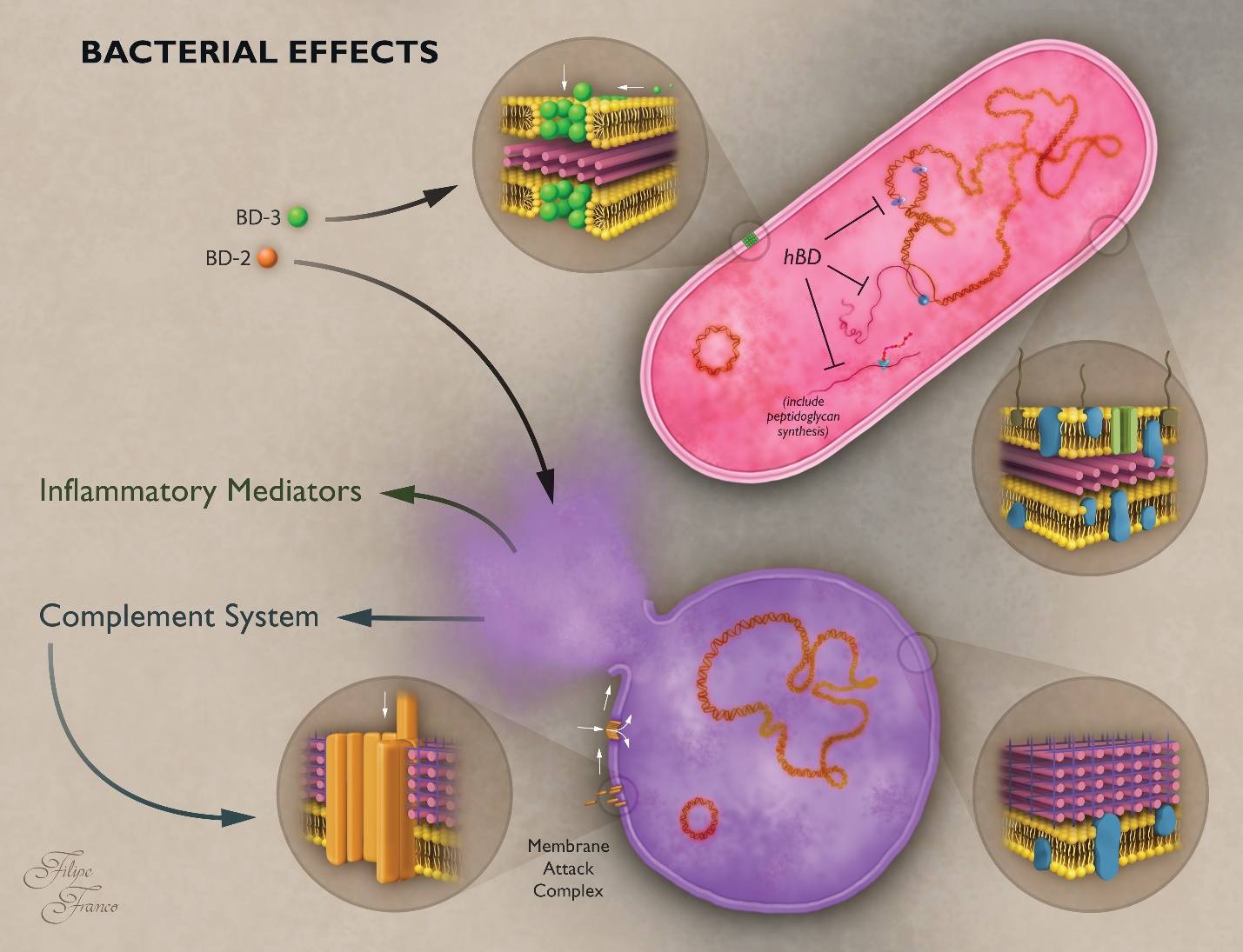
**

**Supplementary Figure 1. Schematic representation of some of the known effects of human β-defensins 2 and 3 on bacteria**. Typical gram-negative bacteria are represented by the pink-red rod. Typical gram-positive bacteria are depicted by the violet coccus. Defensins intercalate into bacterial cell membrane and form pores through bacteria walls promoting osmotic imbalance and bacterial membrane rupture. This, in turn, promotes activation of the alternative pathway of the complement system leading to the formation of membrane attack complexes that further promote bacterial lysis and complement activation. Byproducts of complement activation and bacterial cell rupture act as chemotactic factors promoting the recruitment of inflammatory cells that will enhance bacterial clearance.^8,88^ Intracellularly, defensins inhibit bacterial DNA replication, transduction and translation, disturbing bacterial homeostasis.

BD-2, human β-defensin 2; BD-3, human β-defensin 3; hBD, human β-defensin

**
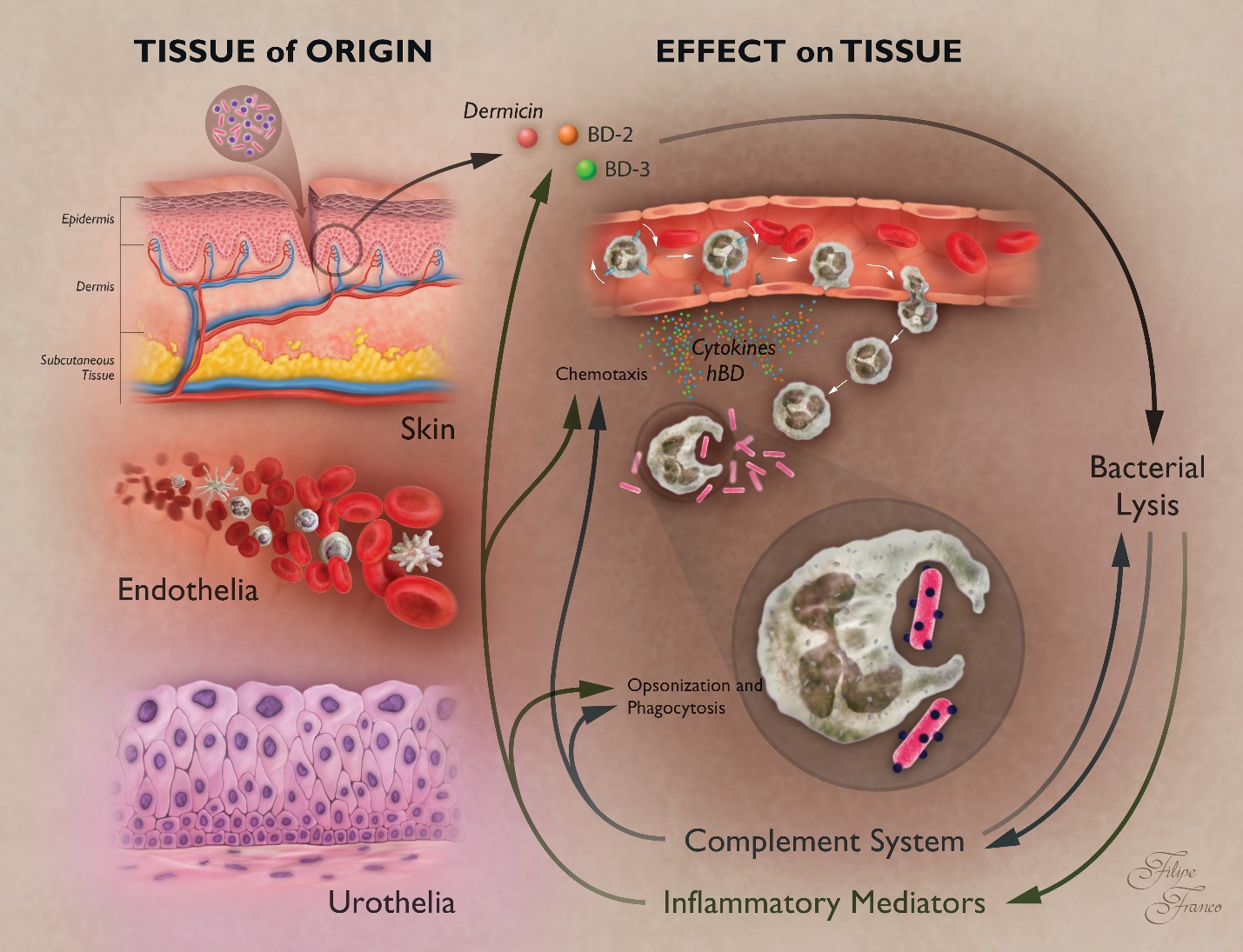
**

**Supplementary Figure 2. Schematic representation of some of the known effects of human β-defensin 2 and 3 on the immune system and their role in the destruction of bacteria.** Gram-negative bacteria are represented by pink-red rods, whereas gram-positive bacteria are depicted by violet cocci.

Antimicrobial peptides, including defensins, are part of the innate resistance against bacterial invasion. Defensins are secreted by multiple epithelia, namely those lining skin, endothelia, gut, airways and the genitourinary tract, as well as by leucocytes and platelets. Microbial components, such as lipopolysaccharide, and other pro-inflammatory stimuli are strong inducers of the expression and secretion of defensins. Defensins, in turn, act as potent chemotactic agents, activate the complement system, promote opsonization and phagocytosis of bacteria, and concomitantly lead to the release of oxygen reactive species and of further pro-inflammatory factors. All these phenomena lead to positive feedback mechanisms that collectively facilitate resolution of bacterial infections.^20,88^

BD-2, human β-defensin2; BD-3, human β-defensin3; hBD, human β-defensin

**Supplementary Tables.**

| **Gene** | **Primer sequence** |
| --- | --- |
| *DEFB4A* | sense – ATGAGGGTCTTGTATCTCCT  *antisense* – TCATGGCTTTTTGCAGCATT |
| *DEFB103A* | sense – TCATGGCTTTTTGCAGCATT  antisense – TTATTTCTTTCTTCGGCAGC |
| *HPRT1* | sense – ATCACATTGTAGCCCTCTGTGTGCTCAAGG  antisense – GTCTGGAATTTCAAATCCAACAAAGTCTGGC |

**Supplementary Table 1.** Primer sequences used to evaluate the expression of beta defensins 2 (*DEFB4A*) and 3 (*DEFB103A*) in cell lines using RT- PCR. The housekeeping gene used as control was hypoxanthine phosphoribosyltransferase 1 (*HPRT1*)*.*

| **Gene** | **Assay ID** |
| --- | --- |
| *DEFB4A* | Hs00175474_m1 |
| *DEFB103A* | Hs00218678_m1 |
| *ACTB* | Rn00667869_m1 |

**Supplementary Table 2.** Assay references used to quantify beta defensins 2 (*DEFB4A*) and 3 (*DEFB103A*) in the rat tissues (flaps) using real-time PCR. The housekeeping gene used as control was actin beta (*ACTB*)*.*

| **Gene** | **Sequence** |
| --- | --- |
| *16S* | sense – GTGSTGCAYGGYTGTCGTCA  *antisense* – ACGTCRTCCMCACCTTCCTC |

**Supplementary Table 3.** Primer sequences used to quantify the number of *Pseudomonas aeruginosa* by real-time PCR. 16S, 16S ribosomal RNA.
